# Supplementary material for: Base-Mediated Claisen Rearrangement of CF3-Containing Bisallyl Ethers
Source: Molecules. 2021 Jul 19;26(14):4365. doi: 10.3390/molecules26144365 (PMC8304132; doi:10.3390/molecules26144365)
Supplement: Supplementary file 1 [file molecules-26-04365-s001.zip › molecules-1287197-supplementary.pdf]

*Supplementary Materials*

# Base-Mediated Claisen Rearrangement of $CF_3$-Containing Bisallyl Ethers

**Yoko Hamada, Rio Matsunaga, Tomoko Kawasaki-Takasuka and Takashi Yamazaki ***

Division of Applied Chemistry, Institute of Engineering, Tokyo University of Agriculture and Technology, 2-24-16 Nakamachi, Koganei 184-8588, Japan; yokoooooo30pama@gmail.com (Y.H.); rio.futa1115@gmail.com (R.M.); takasuka@cc.tuat.ac.jp (T.K.-T.)

* Correspondence: tyamazak@cc.tuat.ac.jp; Tel.: +81-42-388-7038

1. $^{1}$H and $^{13}$C NMR charts for new compounds S2

1.1. (*E*)-1,1,1-Trifluoro-2,4-diphenyl-4-{(prop-2-en-1-yl)oxy}but-2-ene (**3a**) S2

1.2. (*E*)-1,1,1-Trifluoro-2-(4-methoxyphenyl)-4-phenyl-{(prop-2-en-1-yl)oxy}but-2-ene (**3b**) S3

1.3. (*E*)-1,1,1-Trifluoro-2-(4-fluorophenyl)-4-phenyl-{(prop-2-en-1-yl)oxy}but-2-ene (**3c**) S4

1.4. (*E*)-1-Phenyl-1-{(prop-2-en-1-yl)oxy}-3-(trifluoromethyl)pent-2-ene (**3d**) S5

1.5. (*E*)-1,5-Diphenyl-1-{(prop-2-en-1-yl)oxy}-3-(trifluoromethyl)pent-2-ene (**3e**) S6

1.6. (*E*)-1-(4-Methoxyphenyl)-5-phenyl-1-{(prop-2-en-1-yl)oxy}-3-(trifluoromethyl)pent-2-ene (**3f**) S7

1.7. (*E*)-1-(4-Bromophenyl)-5-phenyl-1-{(prop-2-en-1-yl)oxy}-3-(trifluoromethyl)pent-2-ene (**3g**) S8

1.8. (*E*)-4,4,4-Trifluoro-1,3-diphenyl-1-{(prop-2-en-1-yl)oxy}but-1-ene (**4a**) S9

1.9. (*E*)-4,4,4-Trifluoro-3-(4-methoxyphenyl)-1-phenyl-1-{(prop-2-en-1-yl)oxy}but-1-ene (**4b**) S10

1.10. (*E*)-4,4,4-Trifluoro-3-(4-fluorophenyl)-1-phenyl-1-{(prop-2-en-1-yl)oxy}but-1-ene (**4c**) S11

1.11. (*E*)-1-Phenyl-1-{(prop-2-en-1-yl)oxy}-3-(trifluoromethyl)pent-1-ene (**4d**) S12

1.12. (*E*)-1,5-Diphenyl-1-{(prop-2-en-1-yl)oxy}-3-(trifluoromethyl)pent-1-ene (**4e**) S13

1.13. (*E*)-1-(4-Methoxyphenyl)-5-phenyl-1-{(prop-2-en-1-yl)oxy}-3-(trifluoromethyl)pent-1-ene (**4f**) S14/S15

1.14. (*E*)-1-(4-Bromophenyl)-5-phenyl-1-{(prop-2-en-1-yl)oxy}-3-(trifluoromethyl)pent-1-ene (**4g**) S16

1.15. 4,4,4-Trifluoro-1,3-diphenyl-2-(prop-2-en-1-yl)butan-1-one (**5a**) S17

1.16. 4,4,4-Trifluoro-3-(4-methoxyphenyl)-1-phenyl-2-(prop-2-en-1-yl)butan-1-one (**5b**) S18

1.17. 4,4,4-Trifluoro-3-(4-fluorophenyl)-1-phenyl-2- (prop-2-en-1-yl)butan-1-one (**5c**) S19

1.18. 1-Phenyl-2-(prop-2-en-1-yl)-3-(trifluoromethyl)pentan-1-one (**5d**) S20

1.19. 1,5-Diphenyl-2-(prop-2-en-1-yl)-3-(trifluoromethyl)pentan-1-one (**5e**) S21

1.20. 1-(4-Methoxyphenyl)-5-phenyl-2-(prop-2-en-1-yl)-3-(trifluoromethyl)pentan-1-one (**5f**) S22

1.21. 1-(4-Bromophenyl)-5-phenyl-2-(prop-2-en-1-yl)-3-(trifluoromethyl)pentan-1-one (**5g**) S23/S24

## 1. ¹H and ¹³C NMR charts for new compounds

*1.1. (E)-1,1,1-Trifluoro-2,4-diphenyl-4-{(prop-2-en-1-yl)oxy}but-2-ene (3a)*

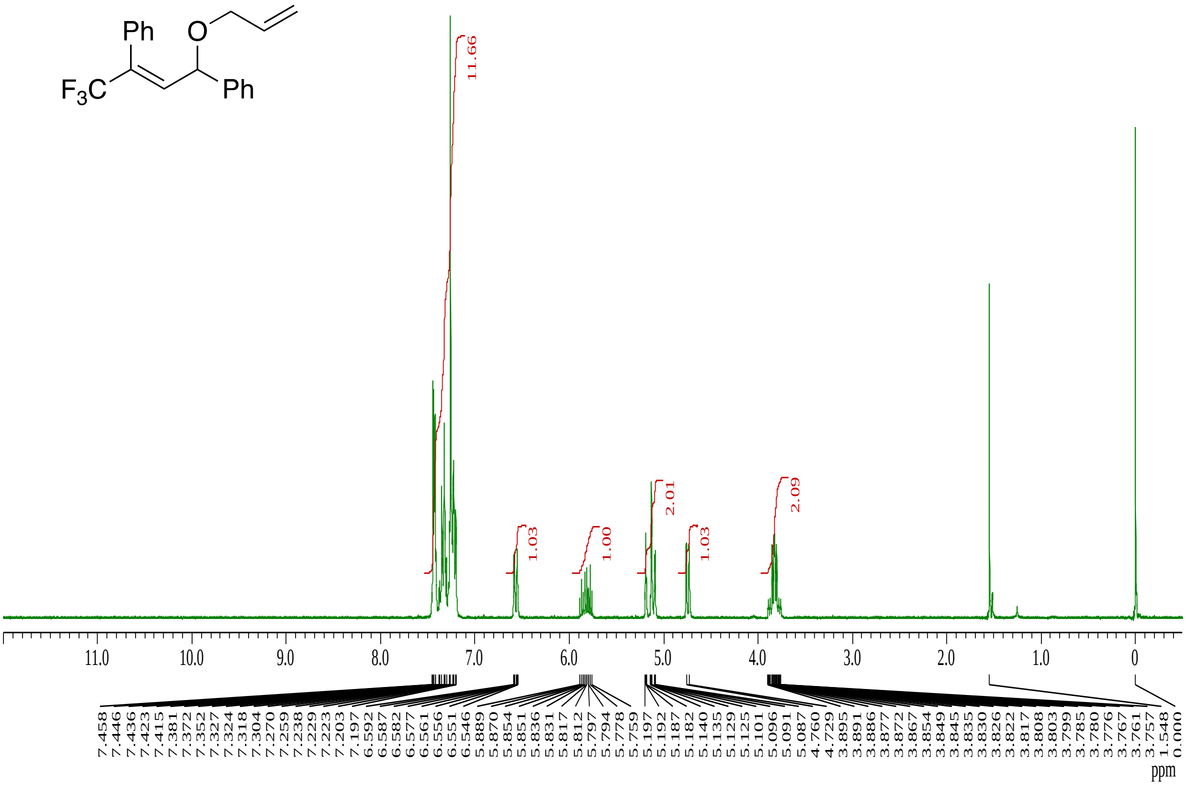


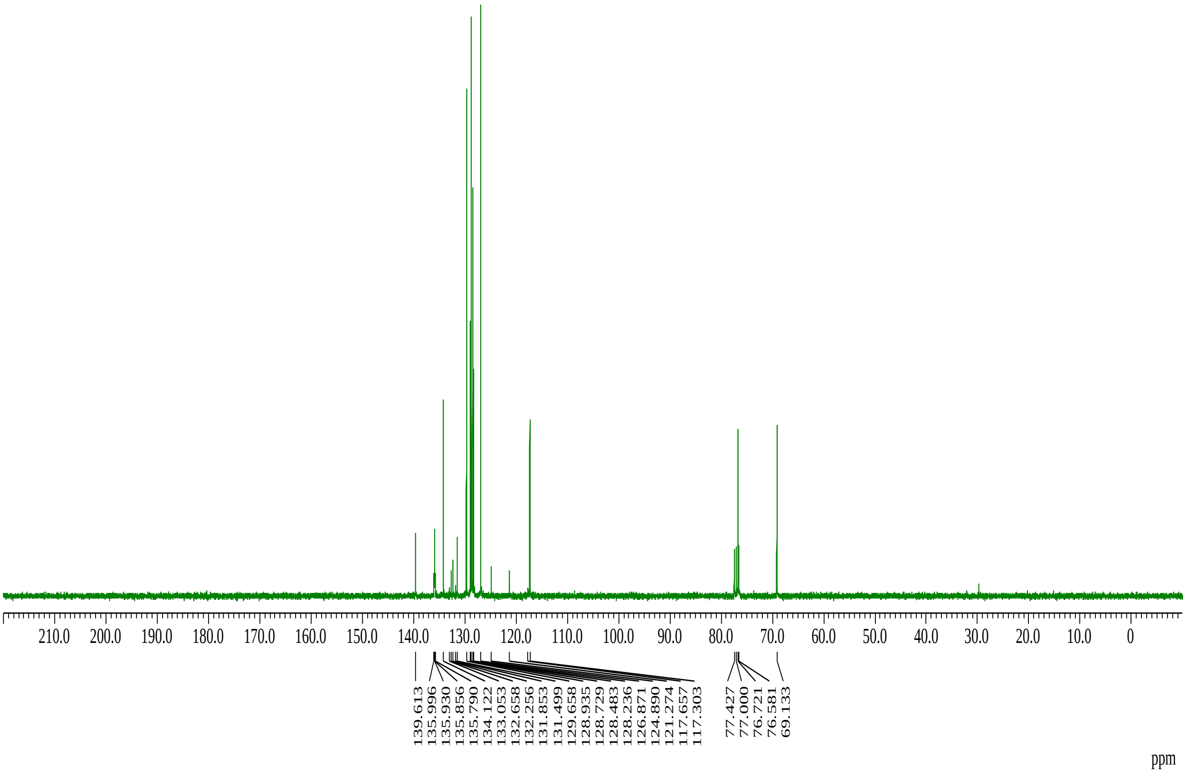

*1.2. (E)-1,1,1-Trifluoro-2-(4-methoxyphenyl)-4-phenyl-{(prop-2-en-1-yl)oxy}but-2-ene (3b)*

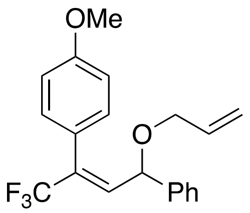


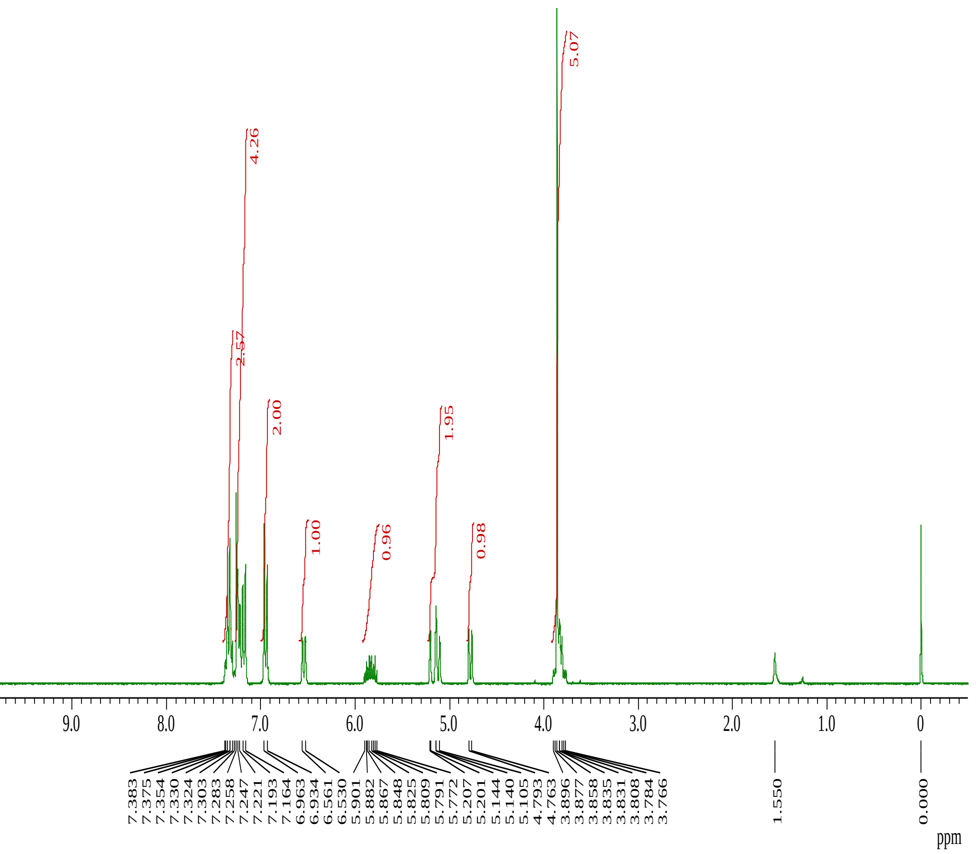

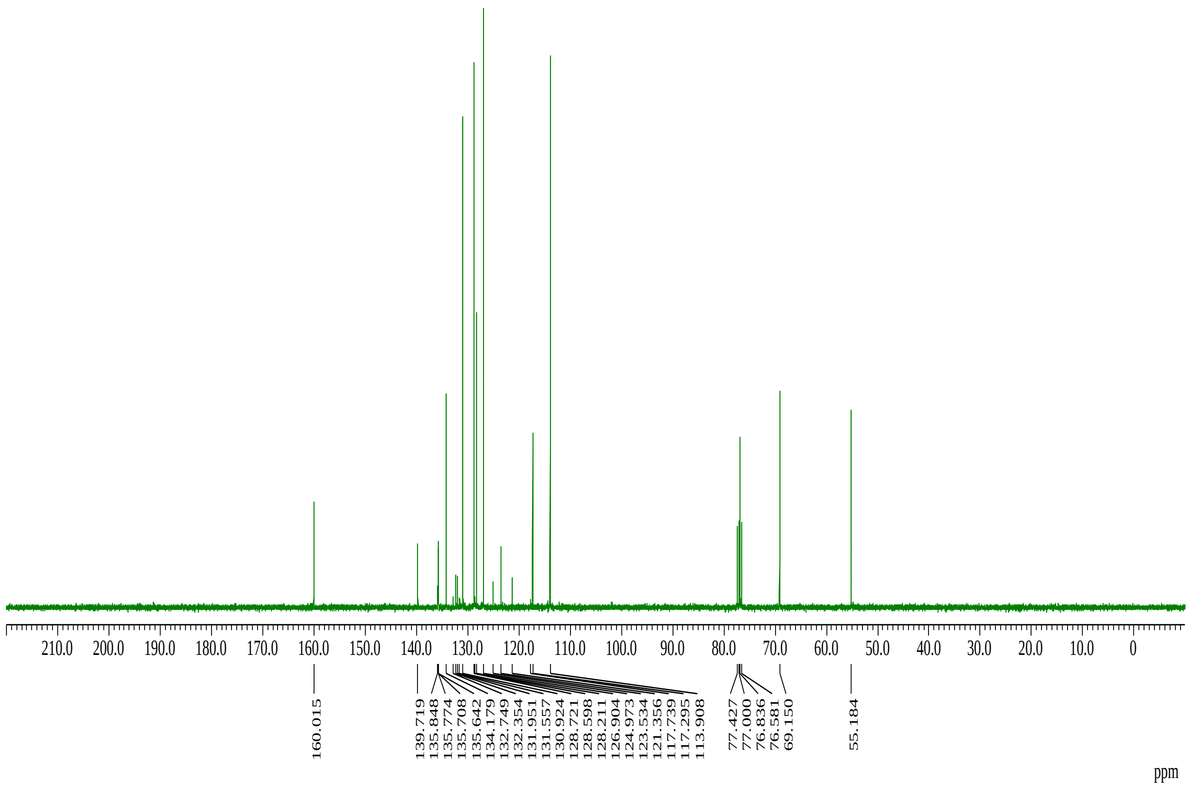

*1.3. (E)-1,1,1-Trifluoro-2-(4-fluorophenyl)-4-phenyl-{(prop-2-en-1-yl)oxy}but-2-ene (3c)*

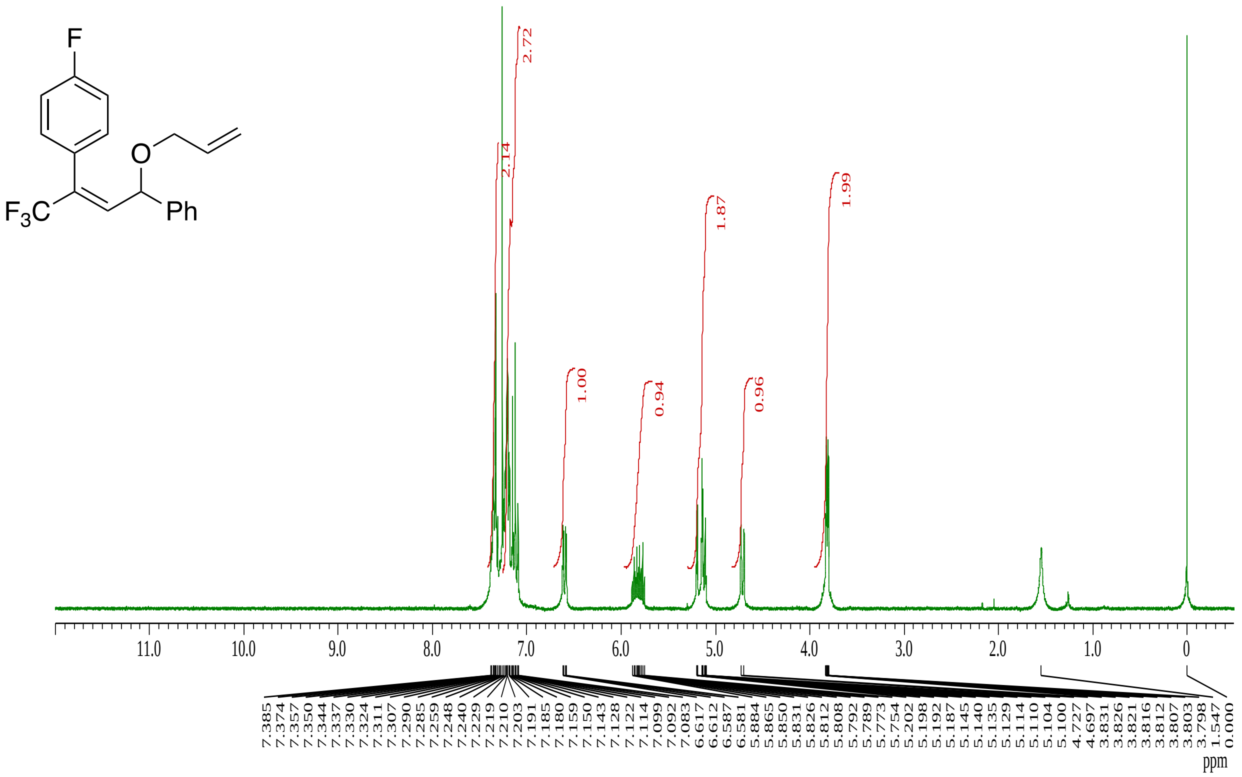


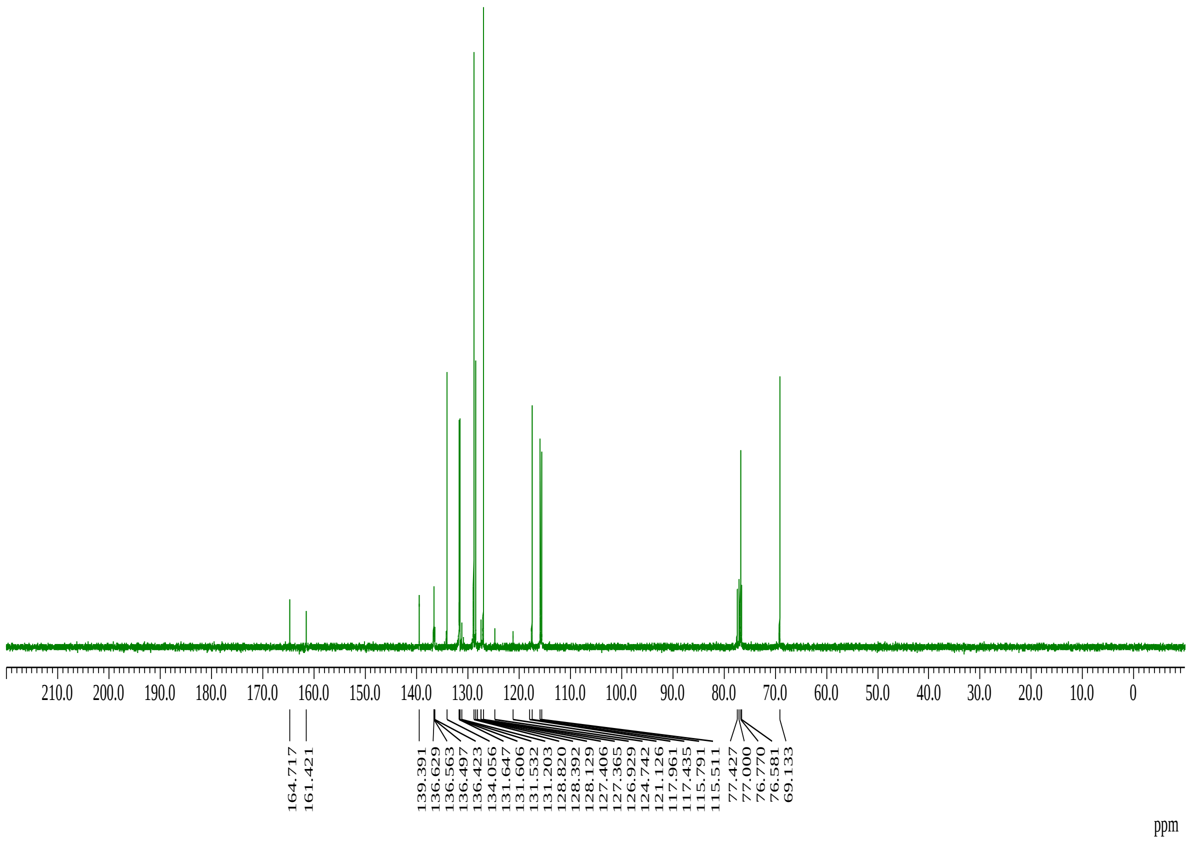

*1.4. (E)-1-Phenyl-1-{(prop-2-en-1-yl)oxy}-3-(trifluoromethyl)pent-2-ene (3d)*

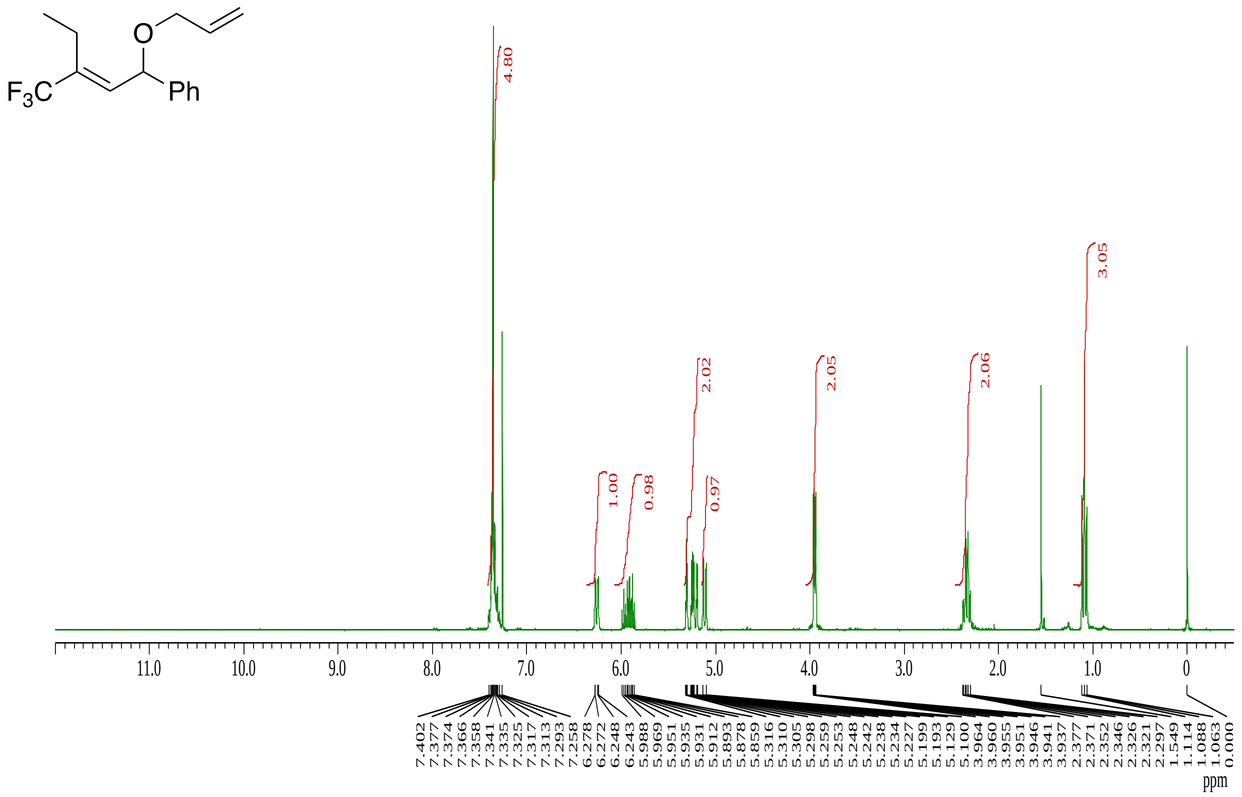


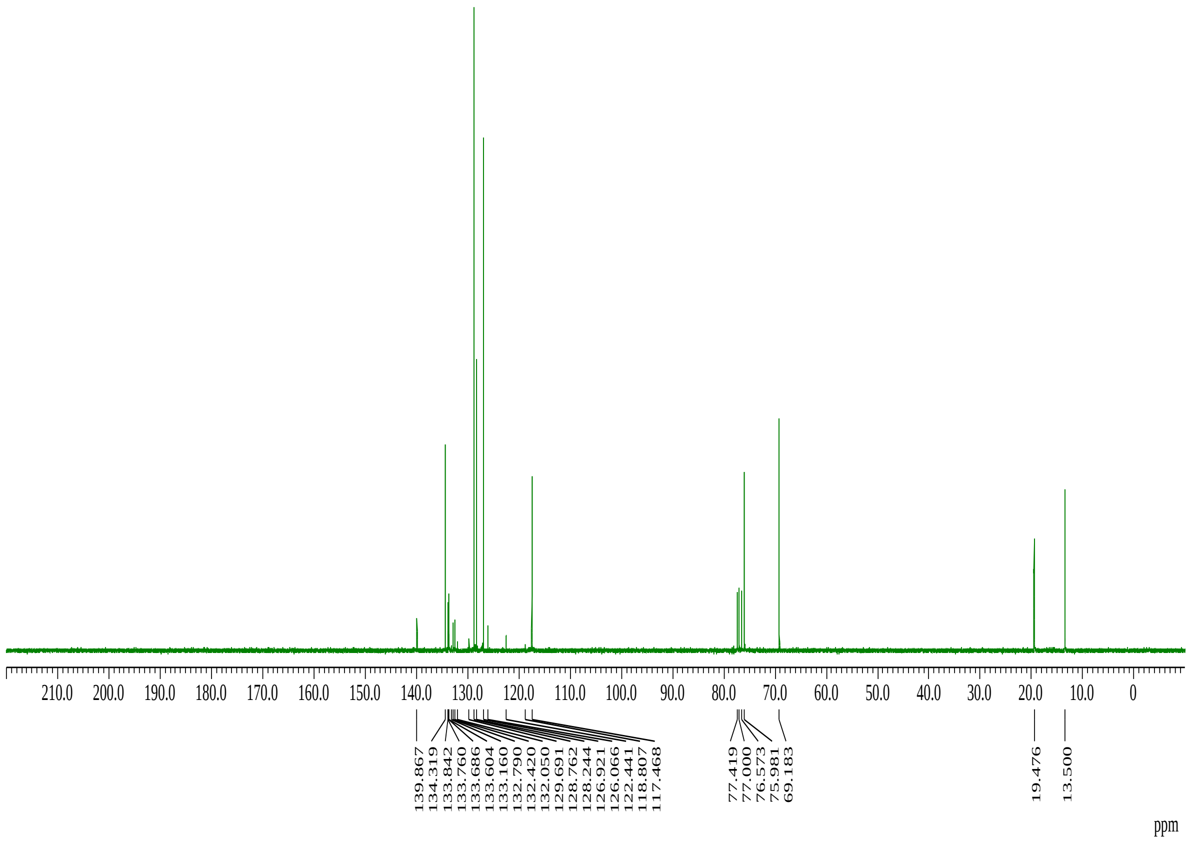

*1.5. (E)-1,5-Diphenyl-1-{(prop-2-en-1-yl)oxy}-3-(trifluoromethyl)pent-2-ene (3e)*

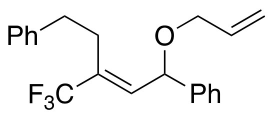


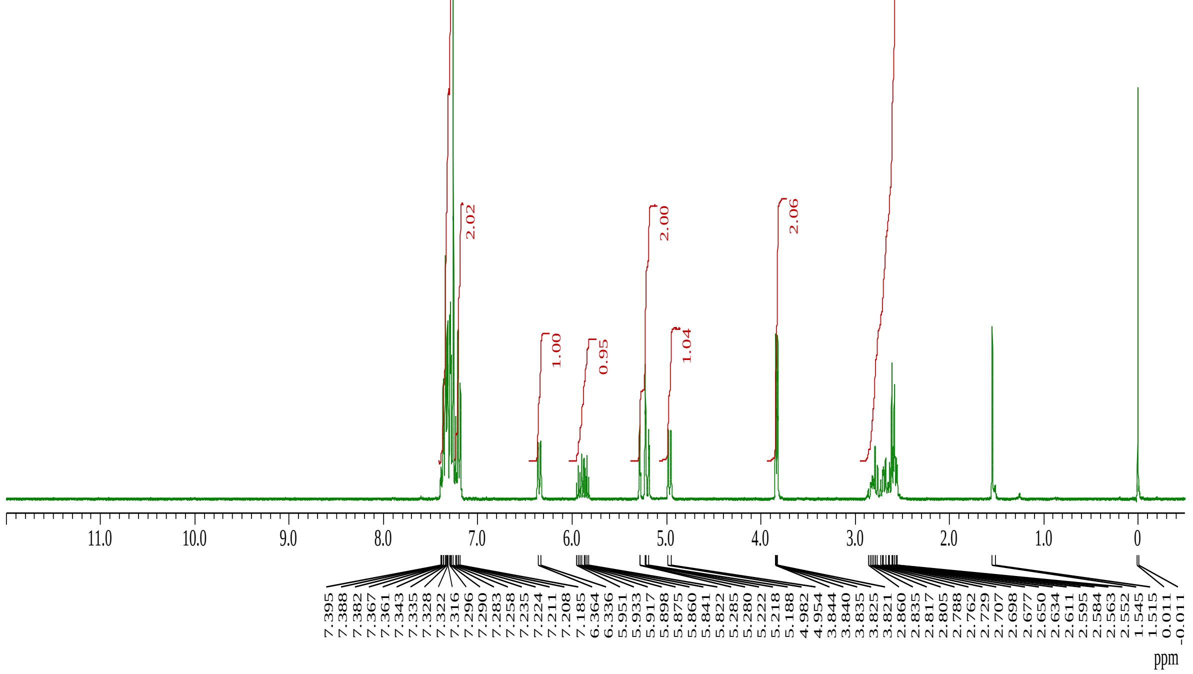


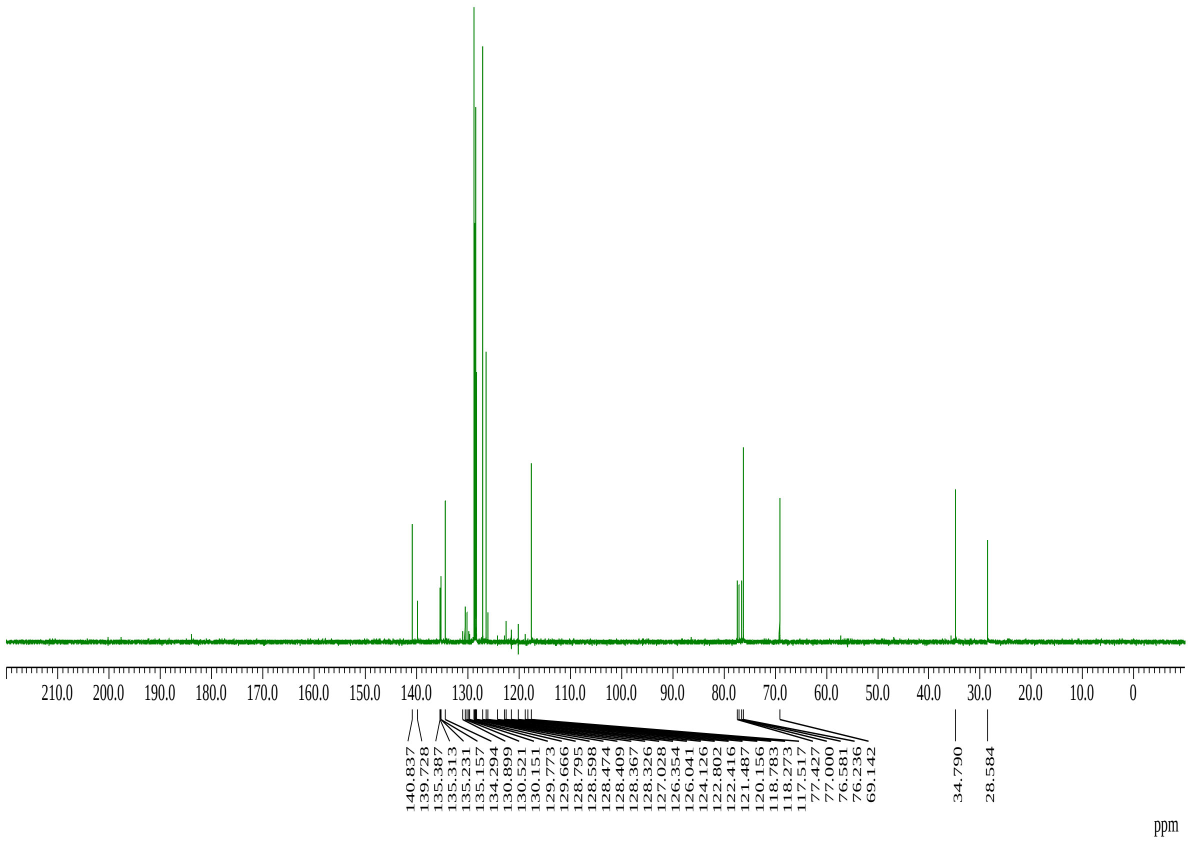

*1.6. (E)-1-(4-Methoxyphenyl)-5-phenyl-1-{(prop-2-en-1-yl)oxy}-3-(trifluoromethyl)pent-2-ene (3f)*

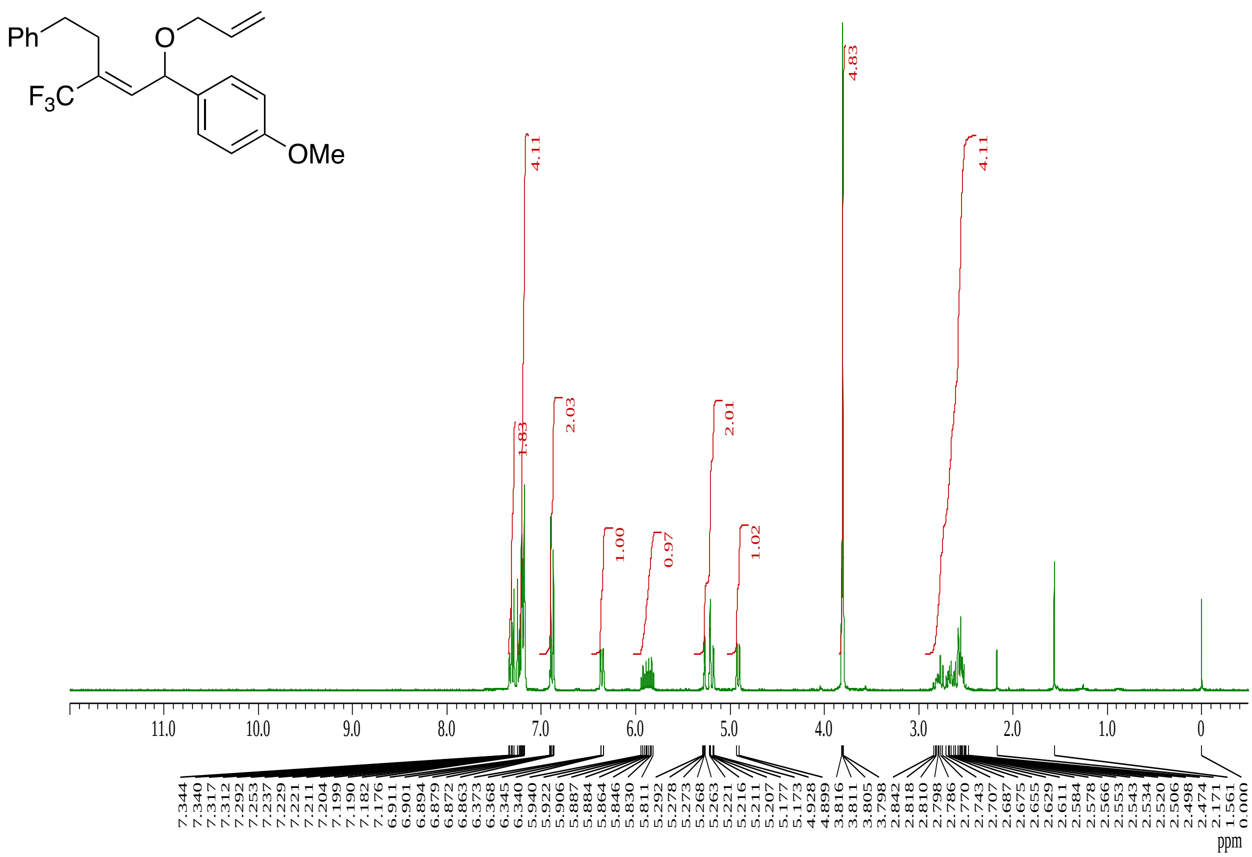

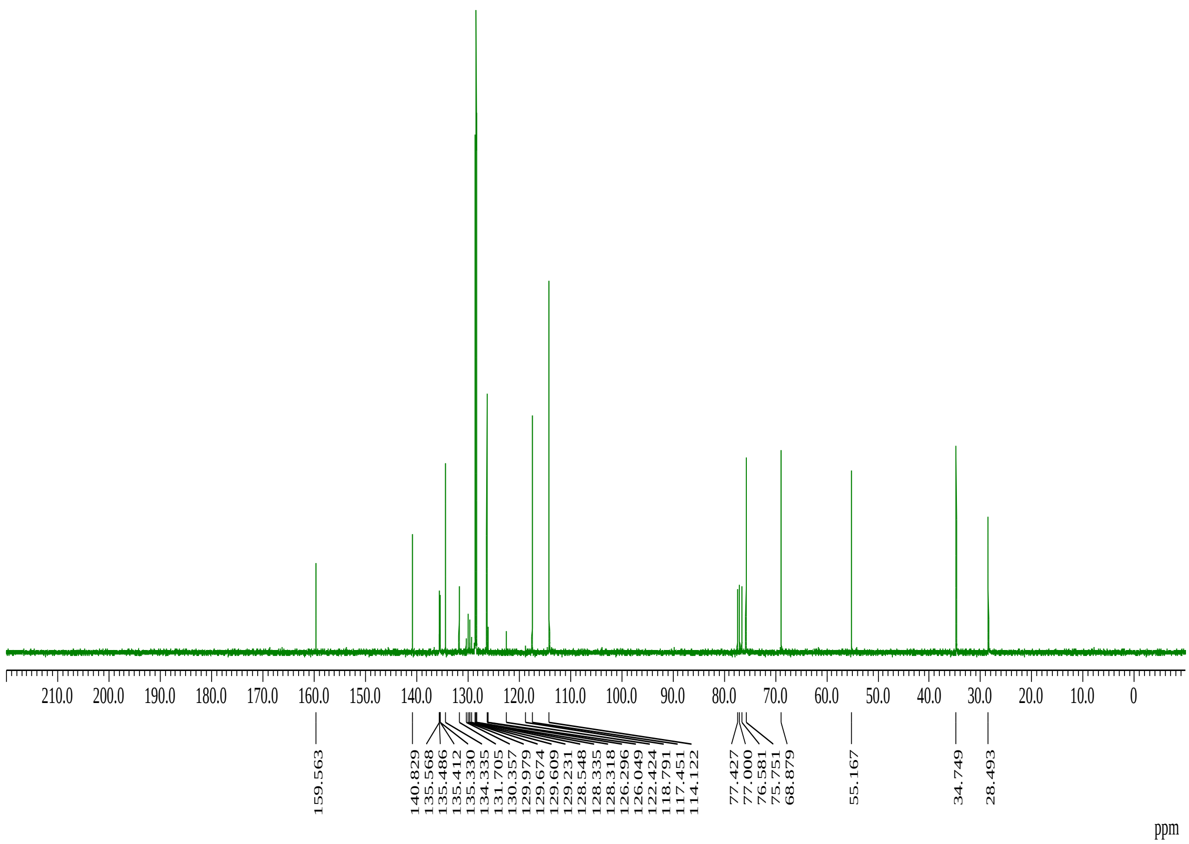


*1.7. (E)-1-(4-Bromophenyl)-5-phenyl-1-{(prop-2-en-1-yl)oxy}-3-(trifluoromethyl)pent-2-ene (3g)*

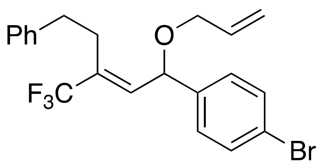


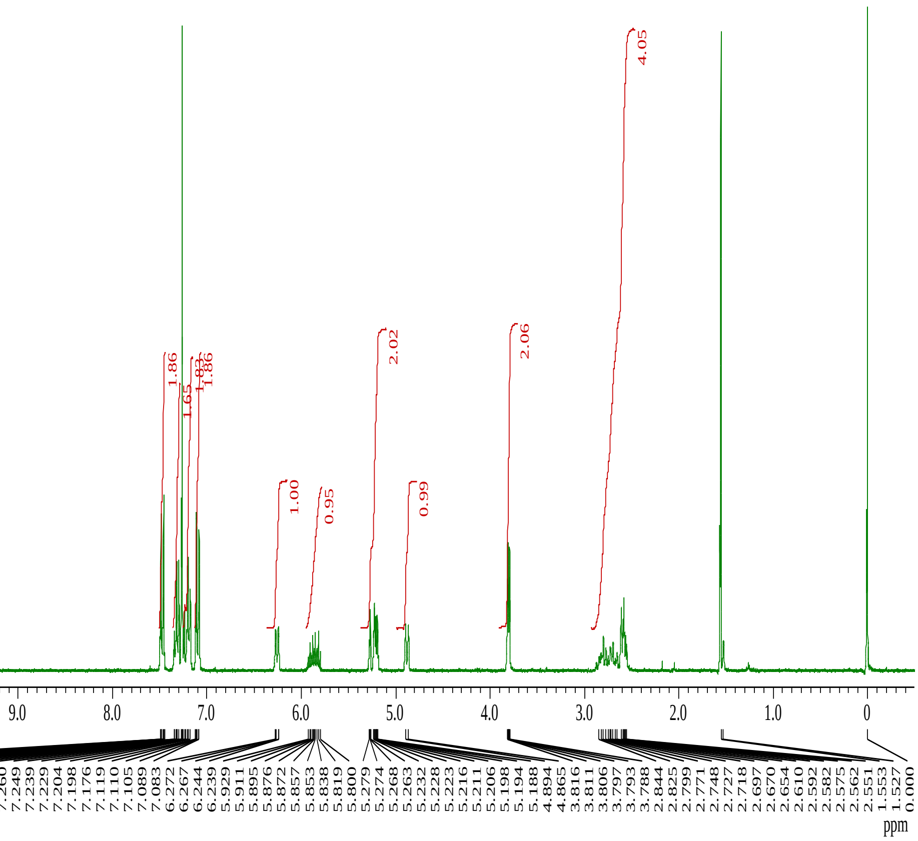

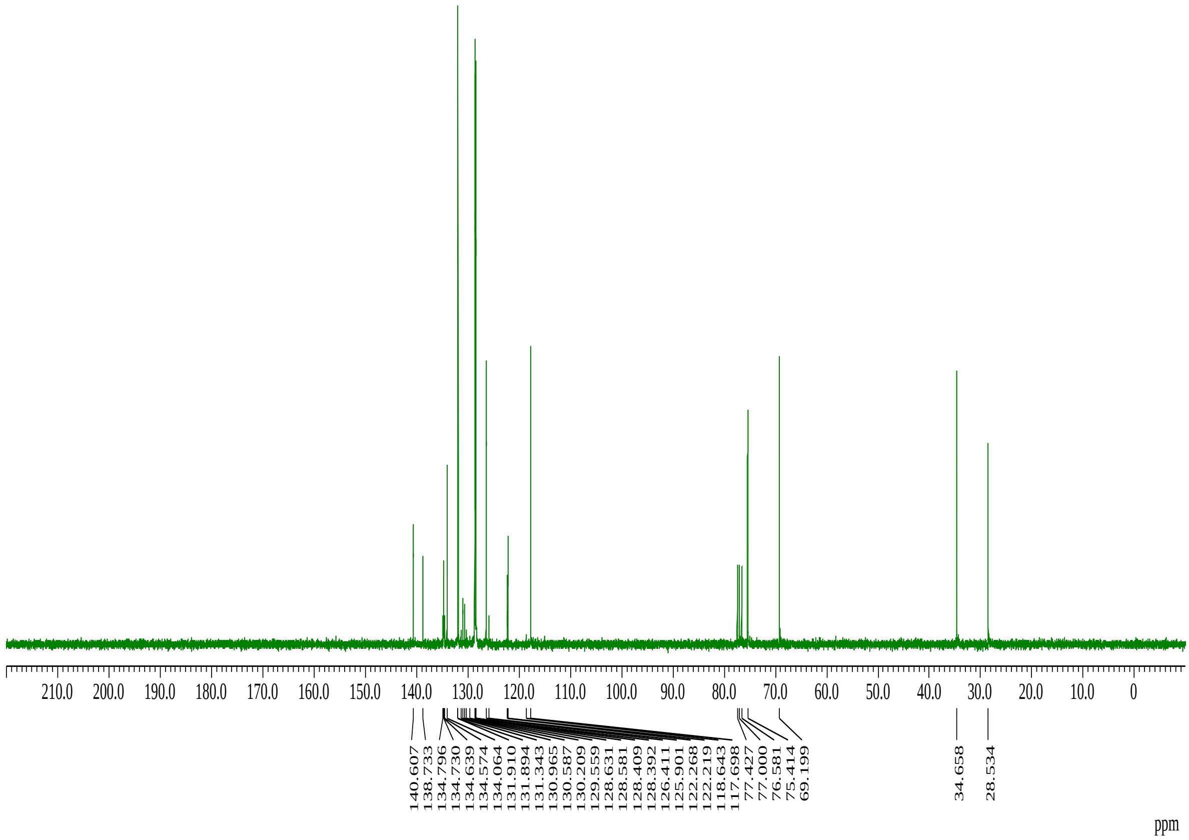


*1.8. (E)-4,4,4-Trifluoro-1,3-diphenyl-1-{(prop-2-en-1-yl)oxy}but-1-ene (4a)*

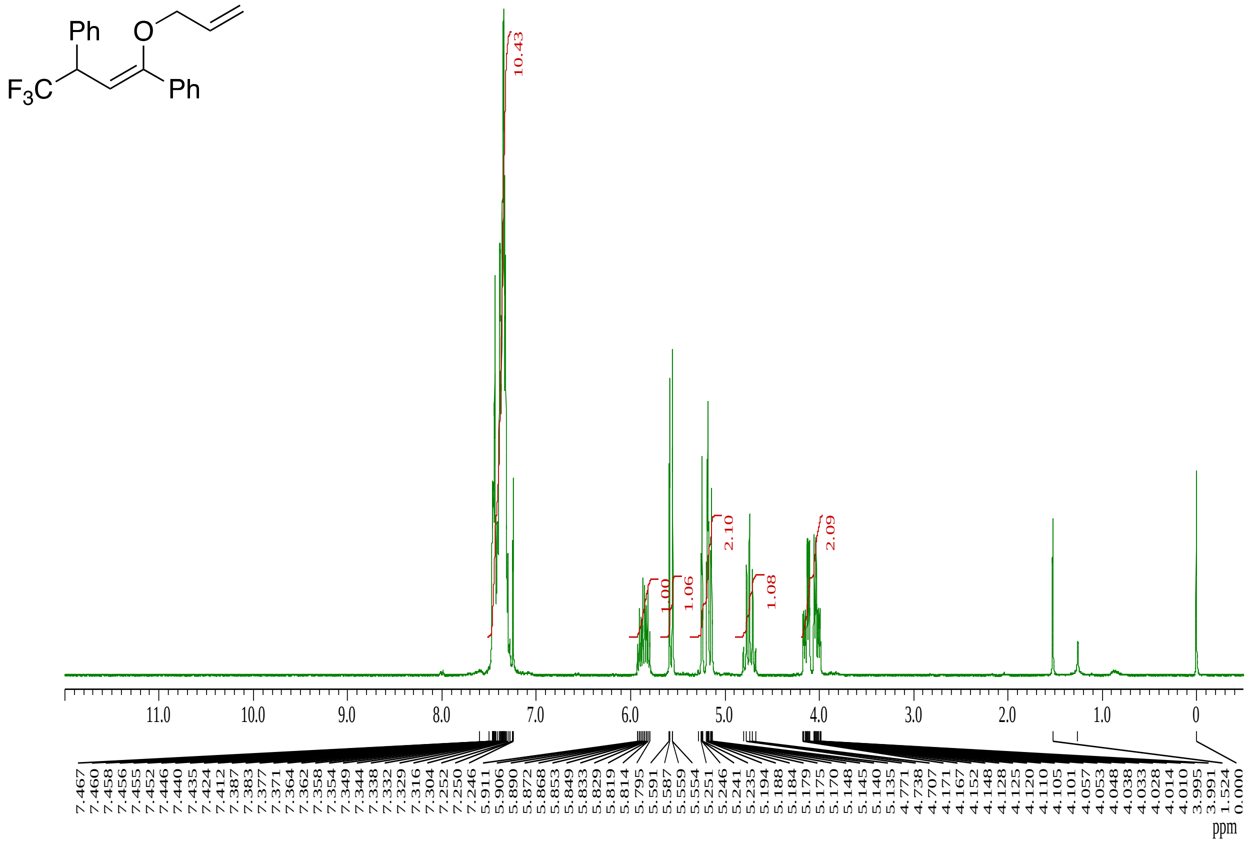

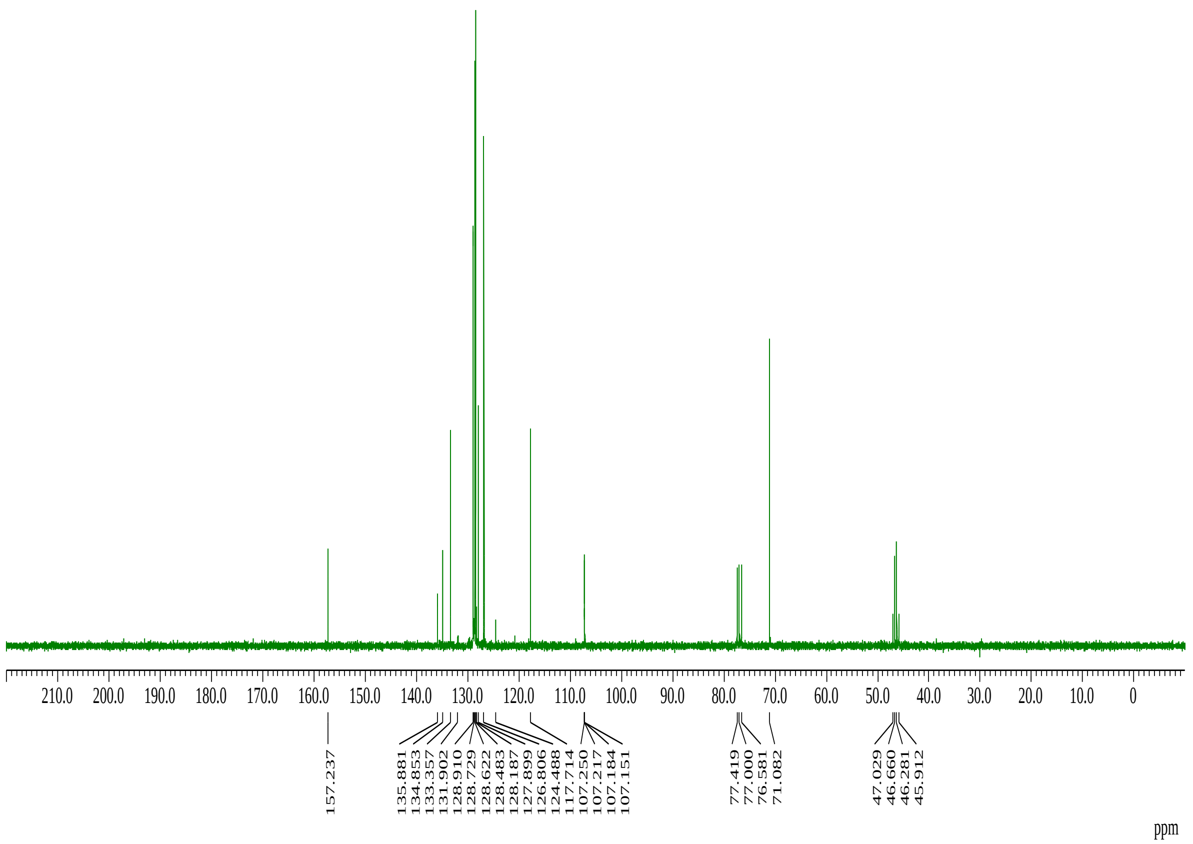


*1.9. (E)-4,4,4-Trifluoro-3-(4-methoxyphenyl)-1-phenyl-1-{(prop-2-en-1-yl)oxy}but-1-ene (4b)*

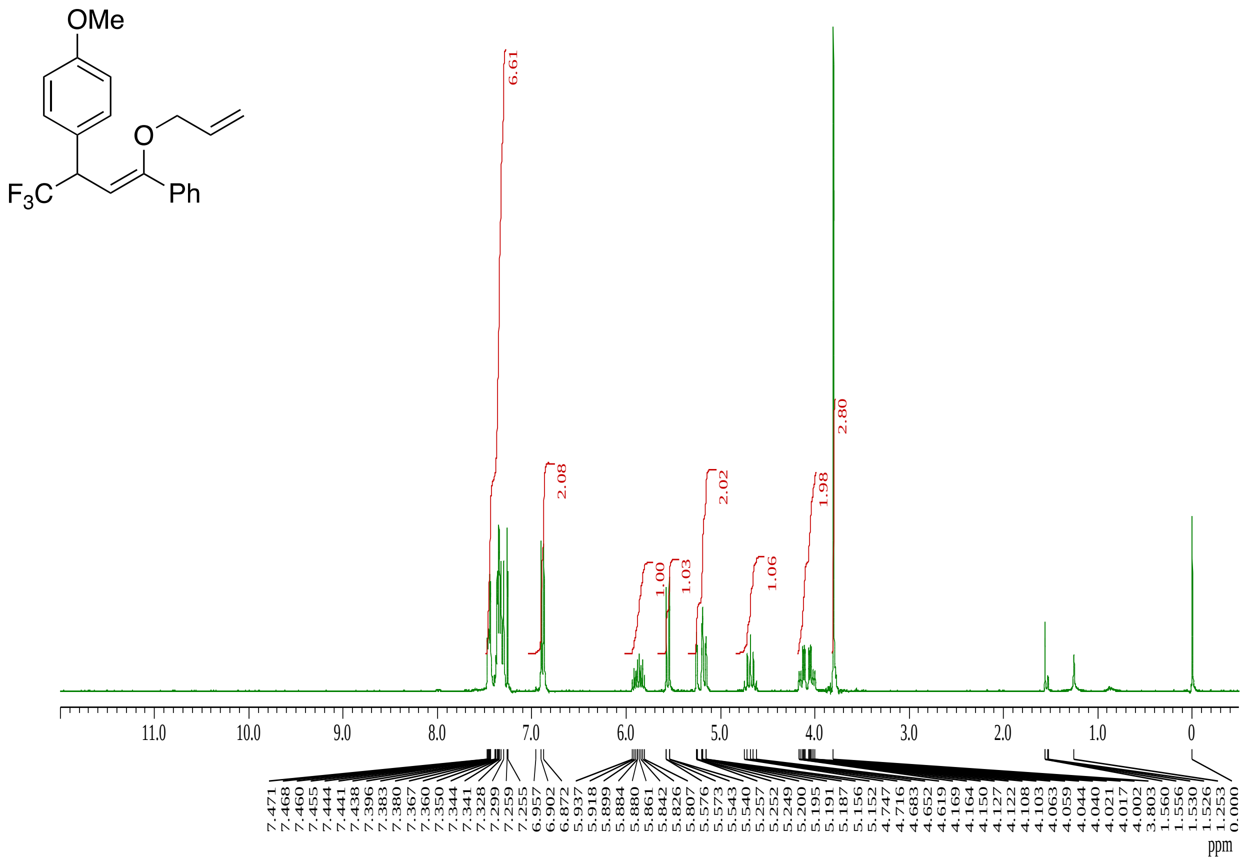

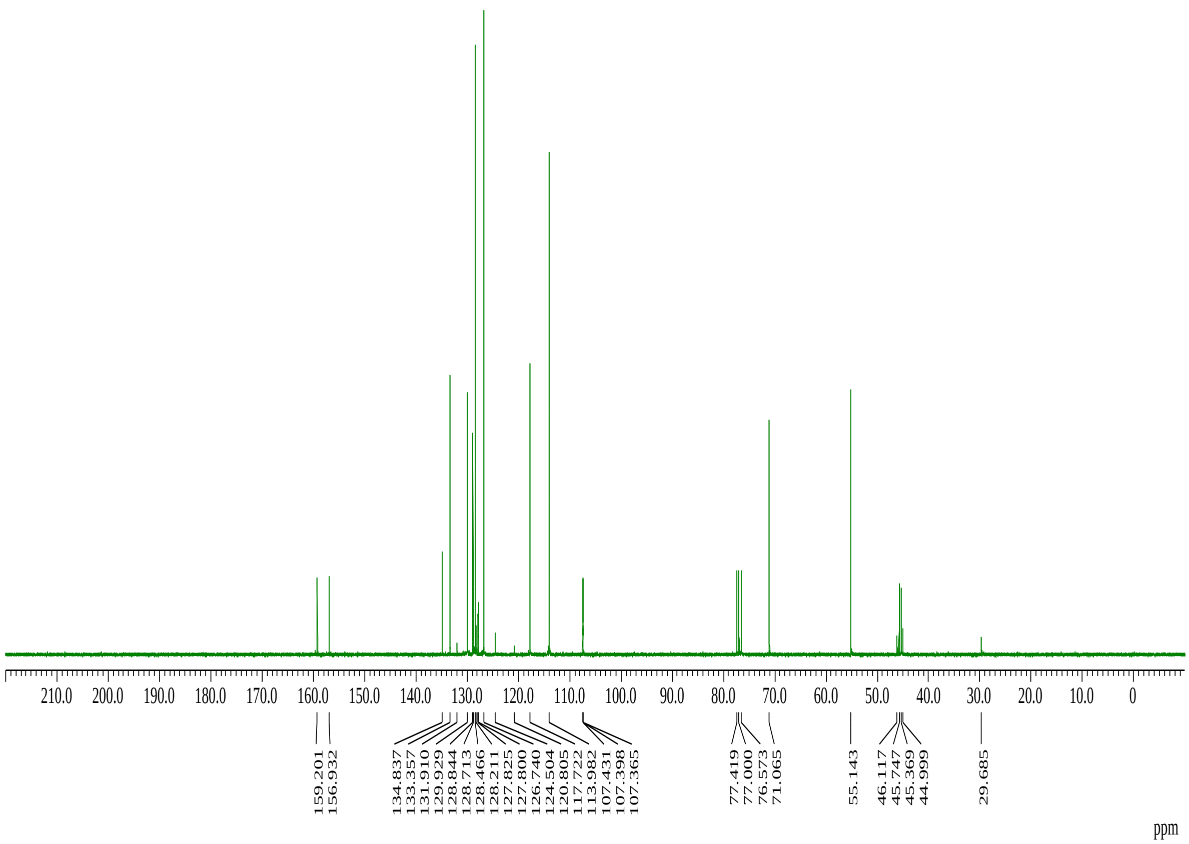

*1.10. (E)-4,4,4-Trifluoro-3-(4-fluorophenyl)-1-phenyl-1-{(prop-2-en-1-yl)oxy}but-1-ene (4c)*

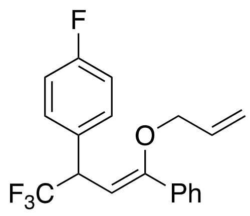


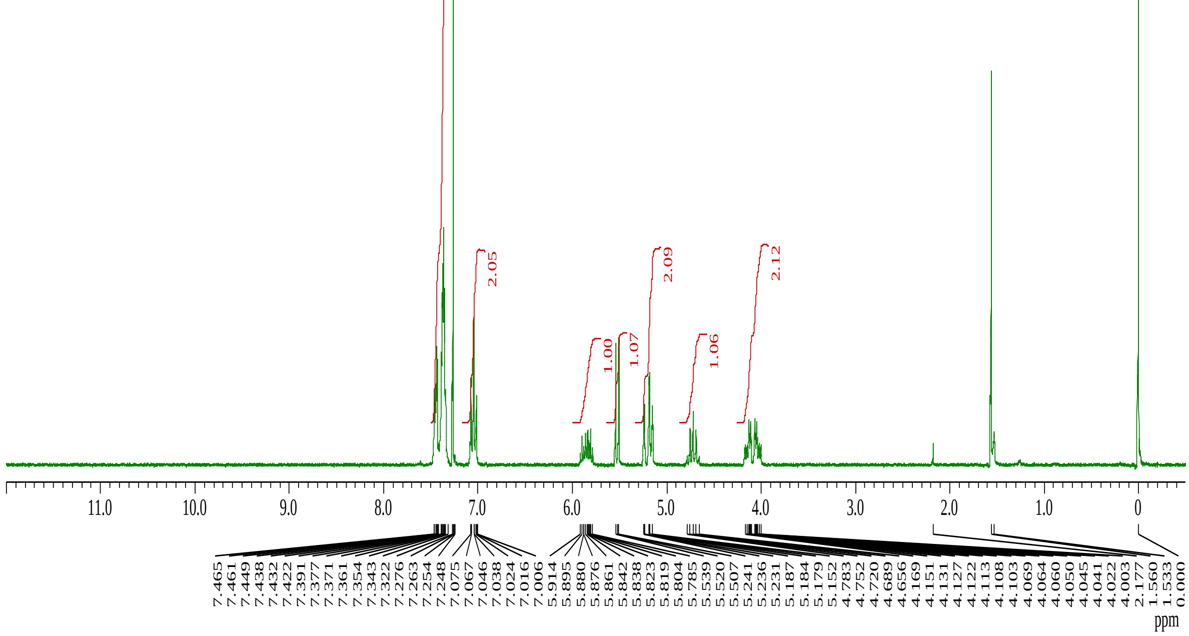

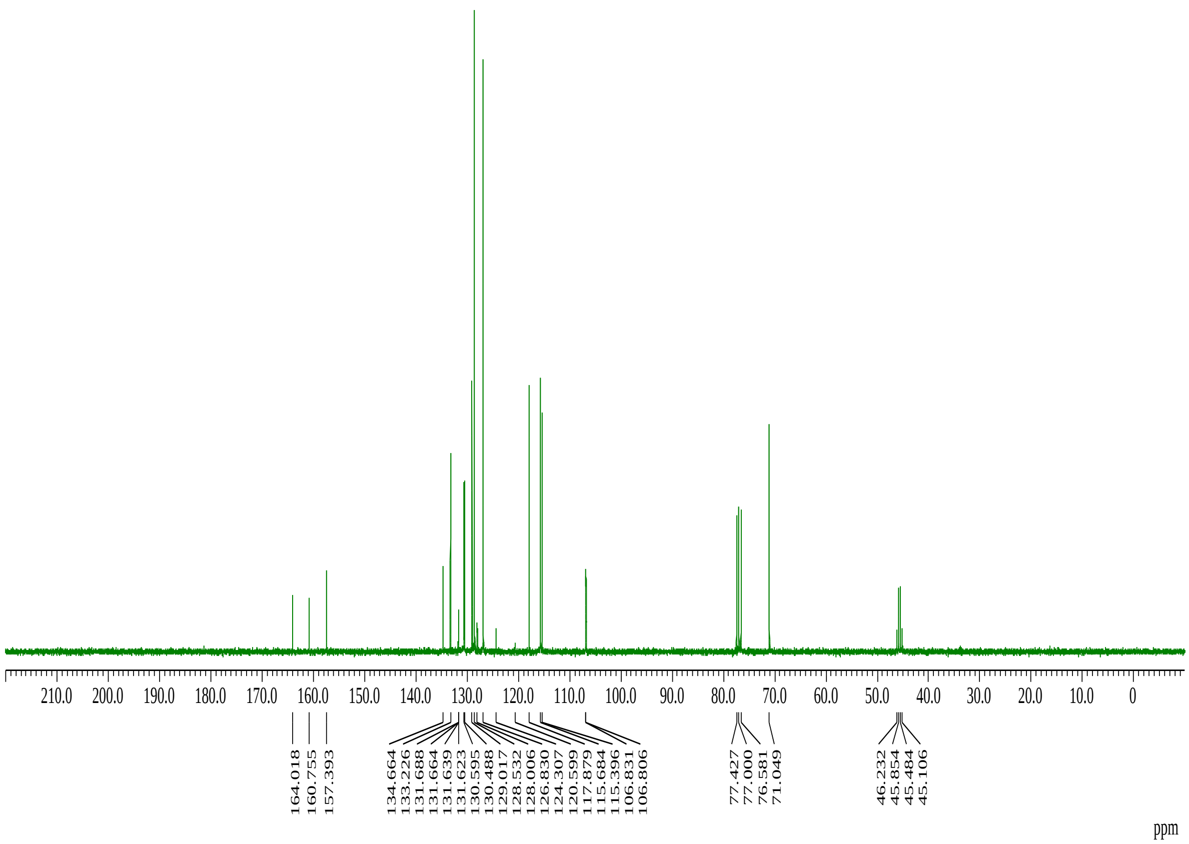


*1.11. (E)-1-Phenyl-1-{(prop-2-en-1-yl)oxy}-3-(trifluoromethyl)pent-1-ene (4d)*

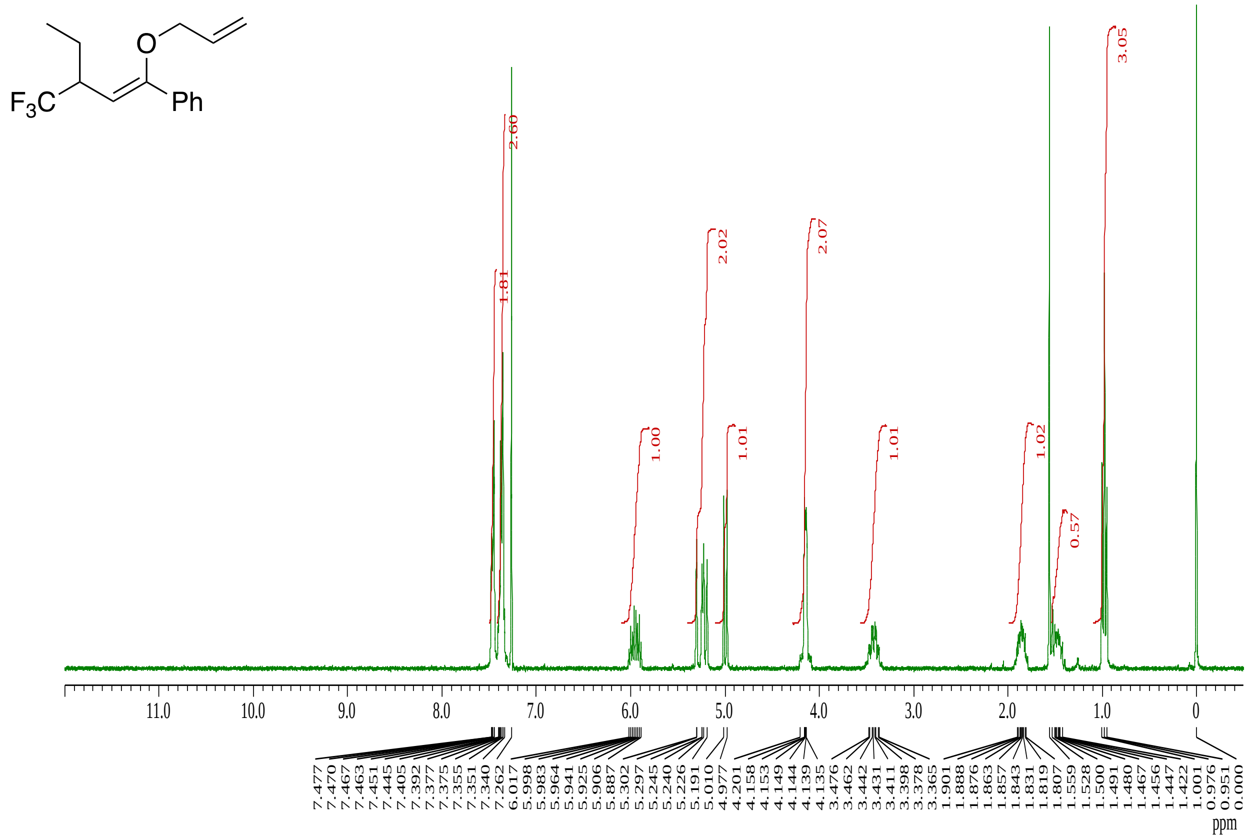

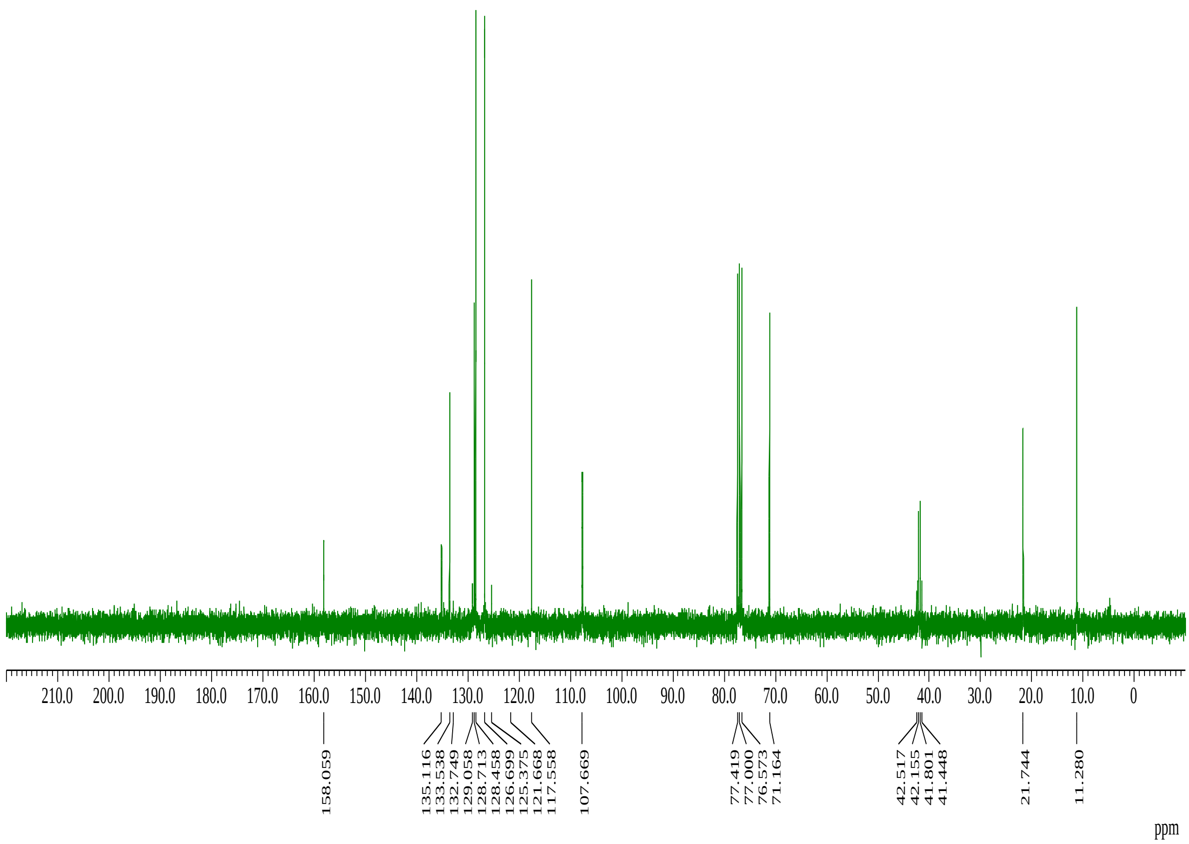


1.12. *(E)-1,5-Diphenyl-1-{(prop-2-en-1-yl)oxy}-3-(trifluoromethyl)pent-1-ene (4e)*

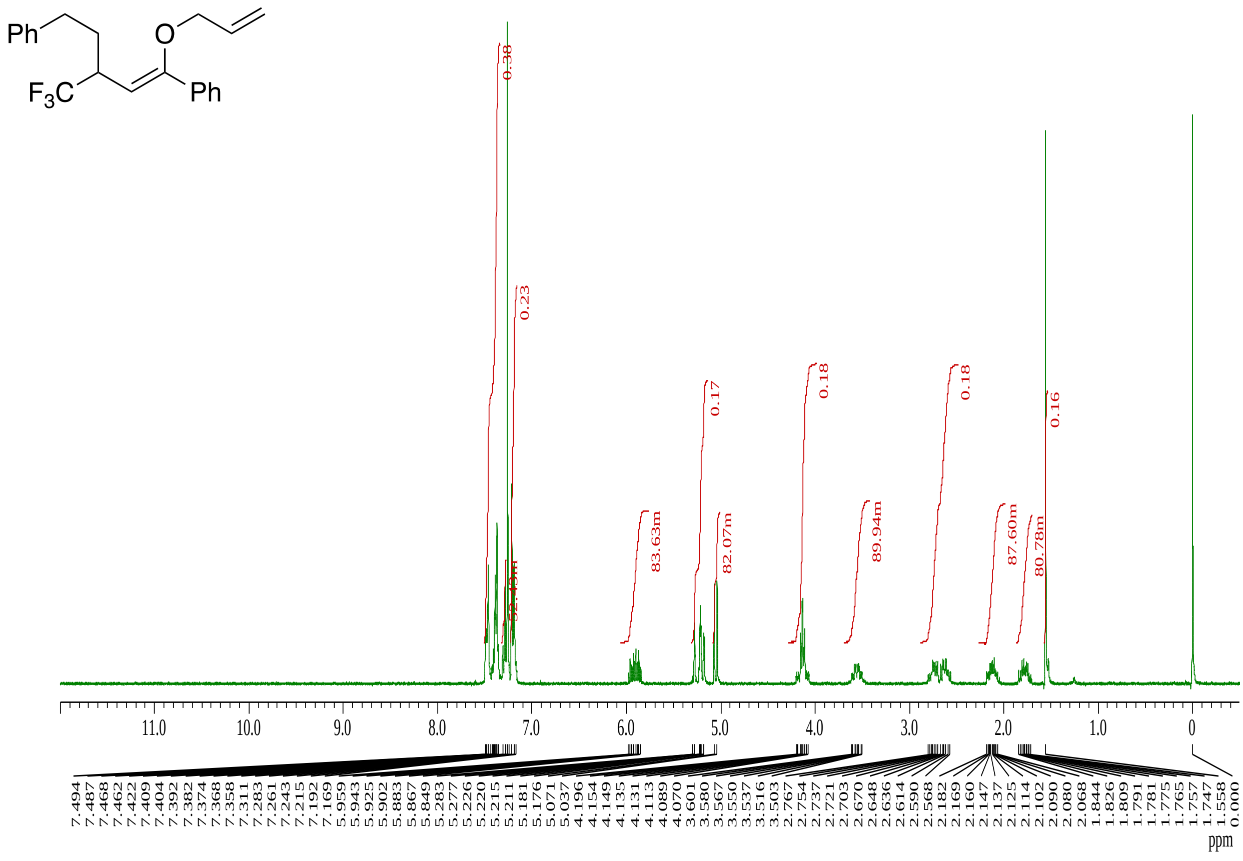

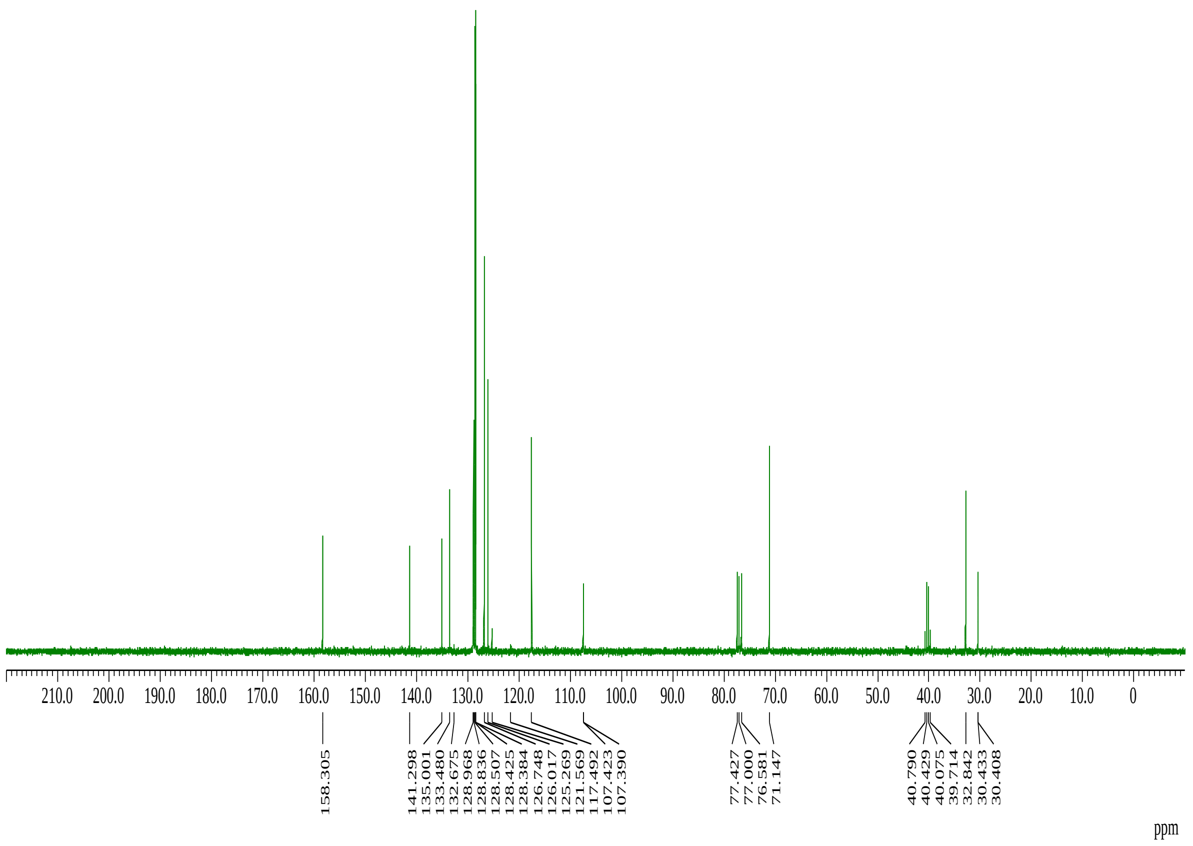


*1.13. (E)-1-(4-Methoxyphenyl)-5-phenyl-1-{(prop-2-en-1-yl)oxy}-3-(trifluoromethyl)pent-1-ene (4f)*

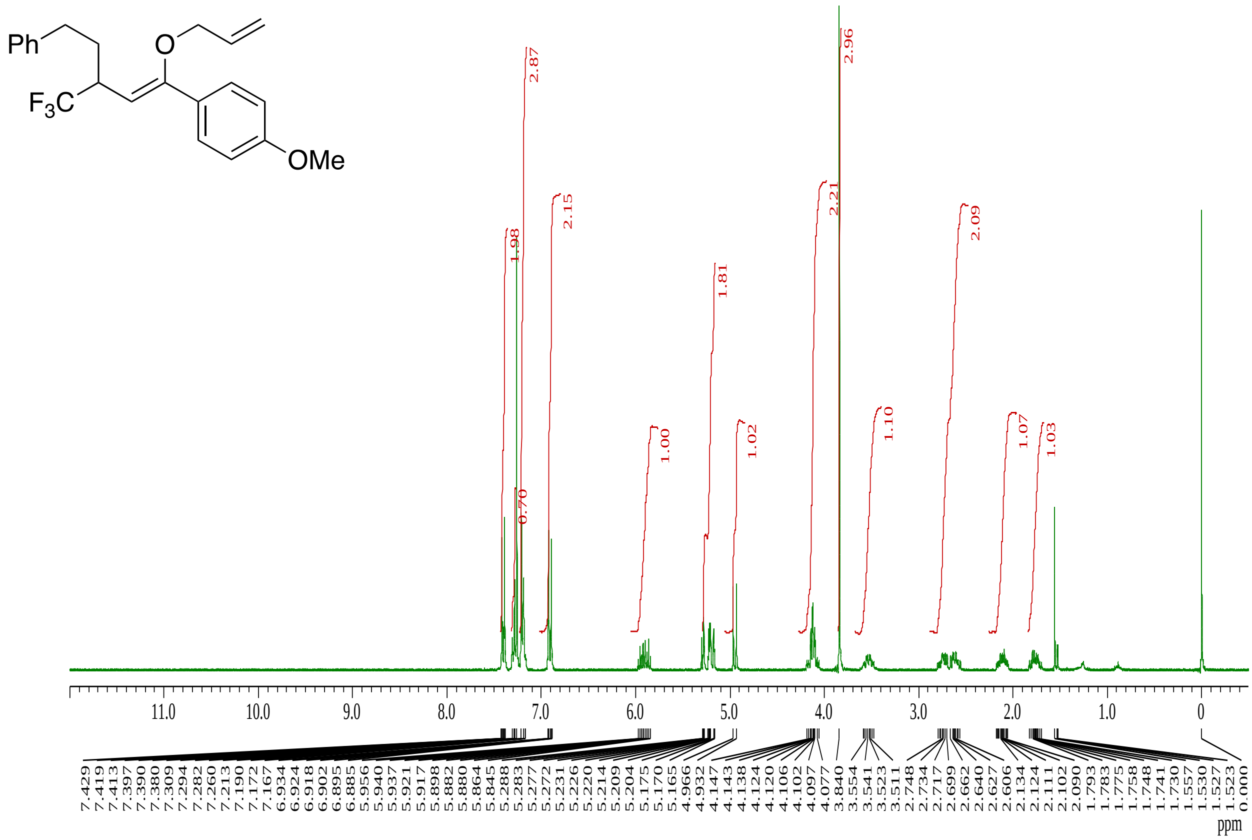

Ph
O
F3C
OMe
2.87
1.98
0.70
2.15
1.00
1.81
1.02
2.21
2.96
1.10
2.09
1.07
1.03
11.0 10.0 9.0 8.0 7.0 6.0 5.0 4.0 3.0 2.0 1.0 0
7.429 7.419 7.413 7.397 7.390 7.380 7.309 7.294 7.282 7.260 7.213 7.190 7.172 7.167 6.934 6.924 6.918 6.902 6.895 6.885 5.956 5.940 5.937 5.921 5.917 5.898 5.882 5.880 5.864 5.845 5.288 5.283 5.277 5.272 5.231 5.226 5.220 5.214 5.209 5.204 5.175 5.170 5.165 4.966 4.932 4.147 4.143 4.138 4.124 4.120 4.106 4.102 4.097 4.077 3.840 3.554 3.541 3.523 3.511 2.748 2.734 2.717 2.699 2.662 2.640 2.627 2.606 2.134 2.124 2.111 2.102 2.090 1.793 1.783 1.775 1.758 1.748 1.741 1.730 1.557 1.530 1.527 1.523 0.000
ppm


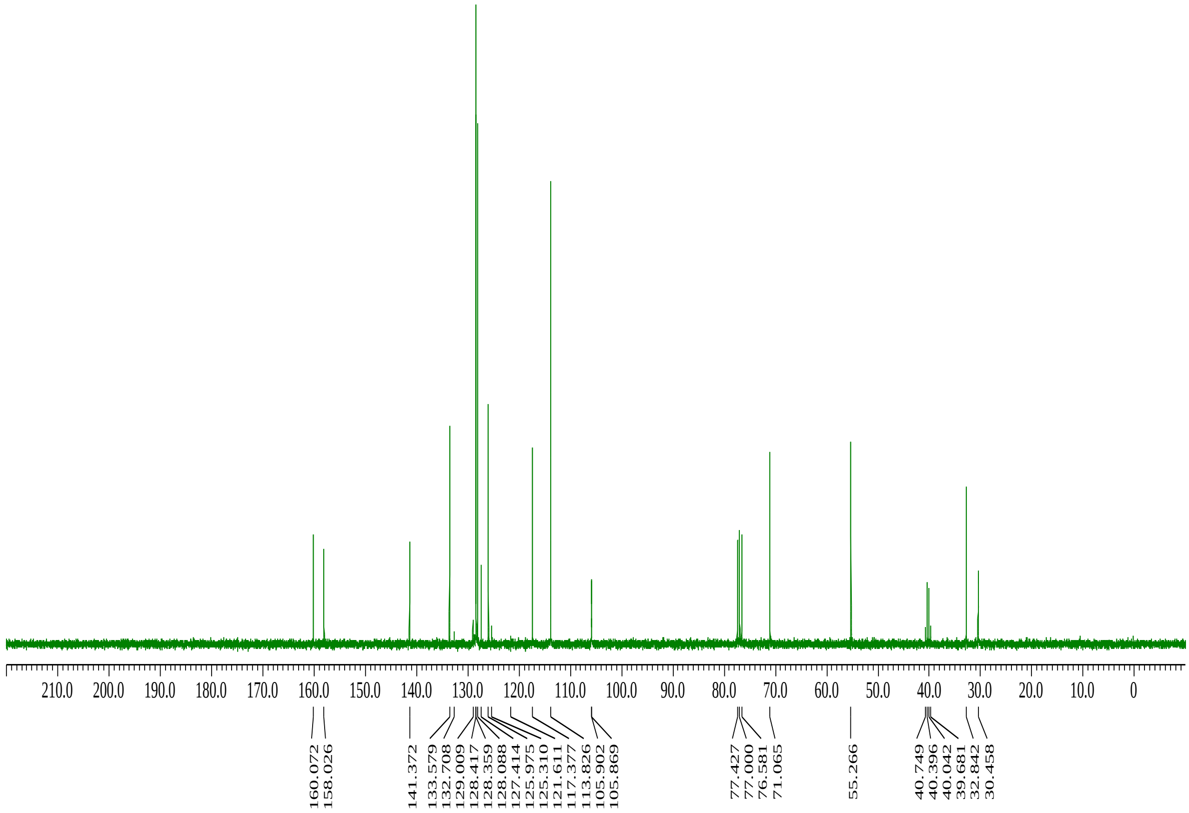

210.0 200.0 190.0 180.0 170.0 160.0 150.0 140.0 130.0 120.0 110.0 100.0 90.0 80.0 70.0 60.0 50.0 40.0 30.0 20.0 10.0 0
160.072 158.026 141.372 133.579 132.708 129.009 128.417 128.359 128.088 127.414 125.975 125.310 121.611 117.377 113.826 105.902 105.869 77.427 77.000 76.581 71.065 55.266 40.749 40.396 40.042 39.681 32.842 30.458
ppm

*1.14. (E)-1-(4-Bromophenyl)-5-phenyl-1-{(prop-2-en-1-yl)oxy}-3-(trifluoromethyl)pent-1-ene (4g)*

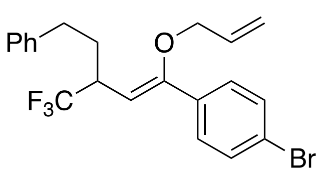


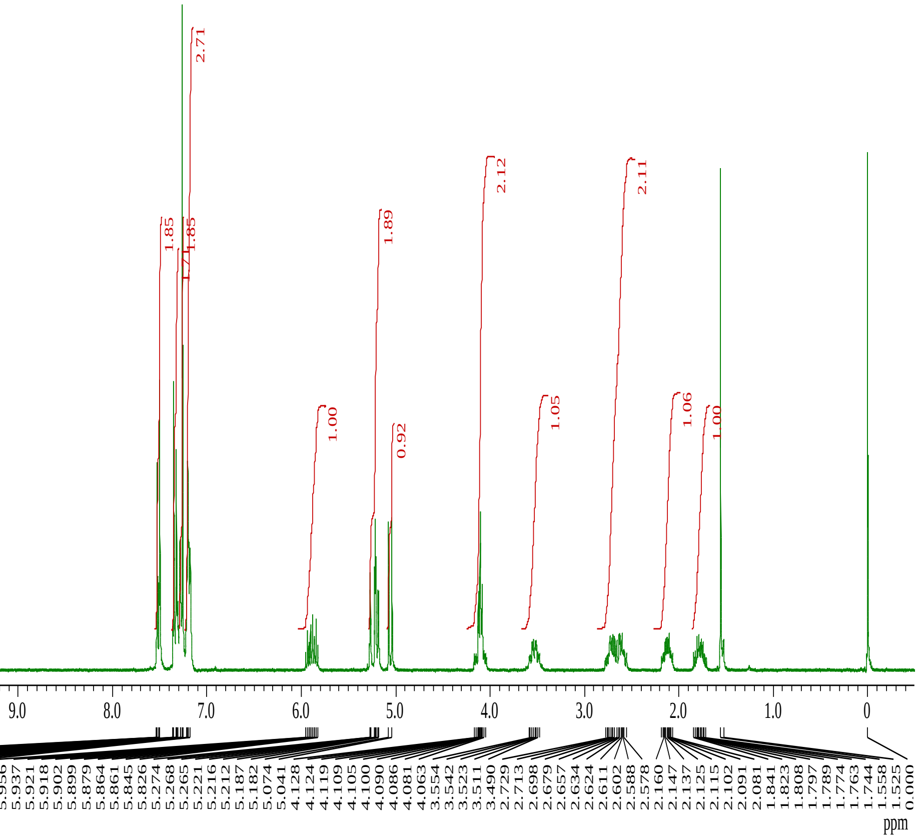

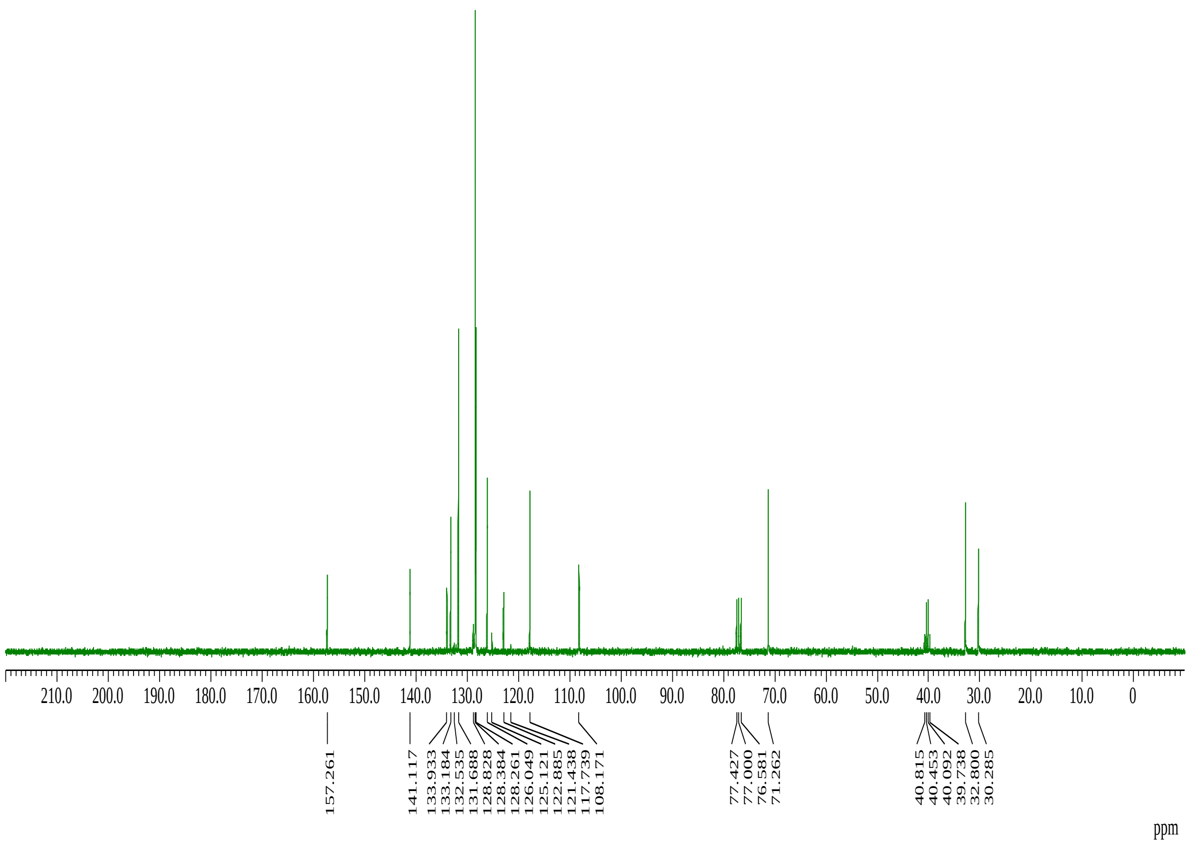


*1.15. 4,4,4-Trifluoro-1,3-diphenyl-2-(prop-2-en-1-yl)butan-1-one (5a)*

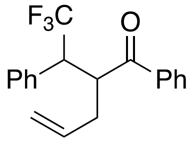


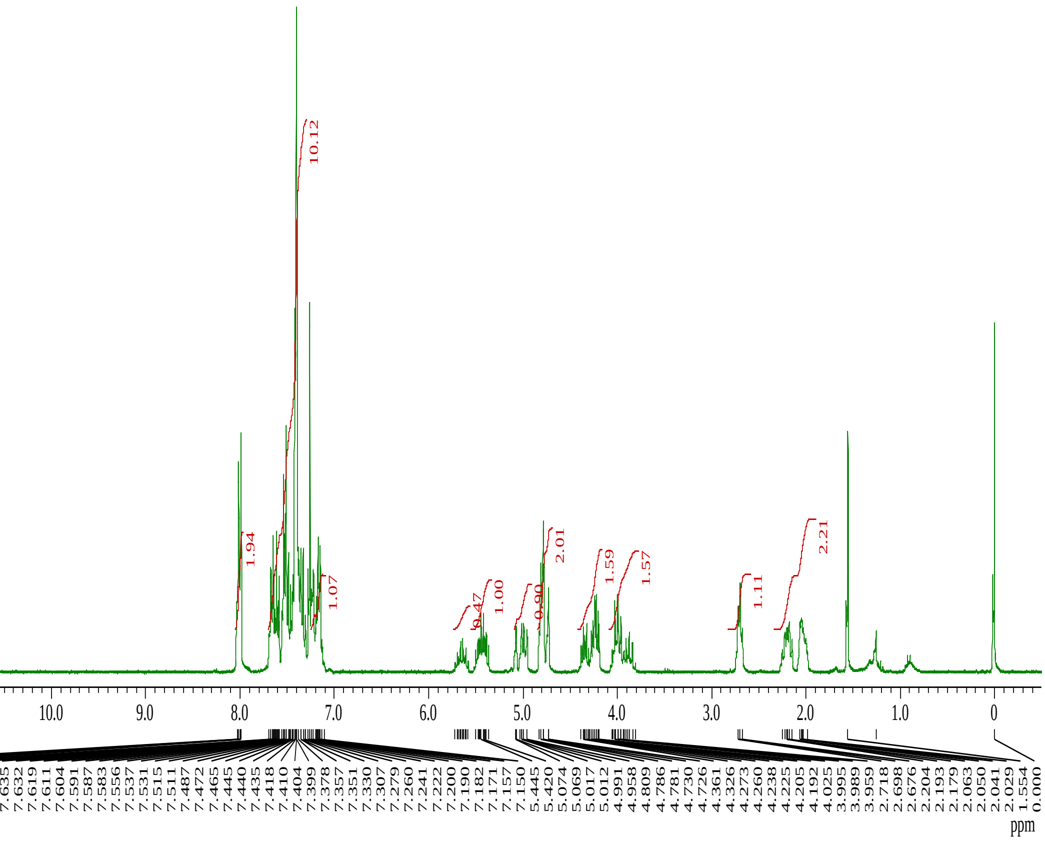

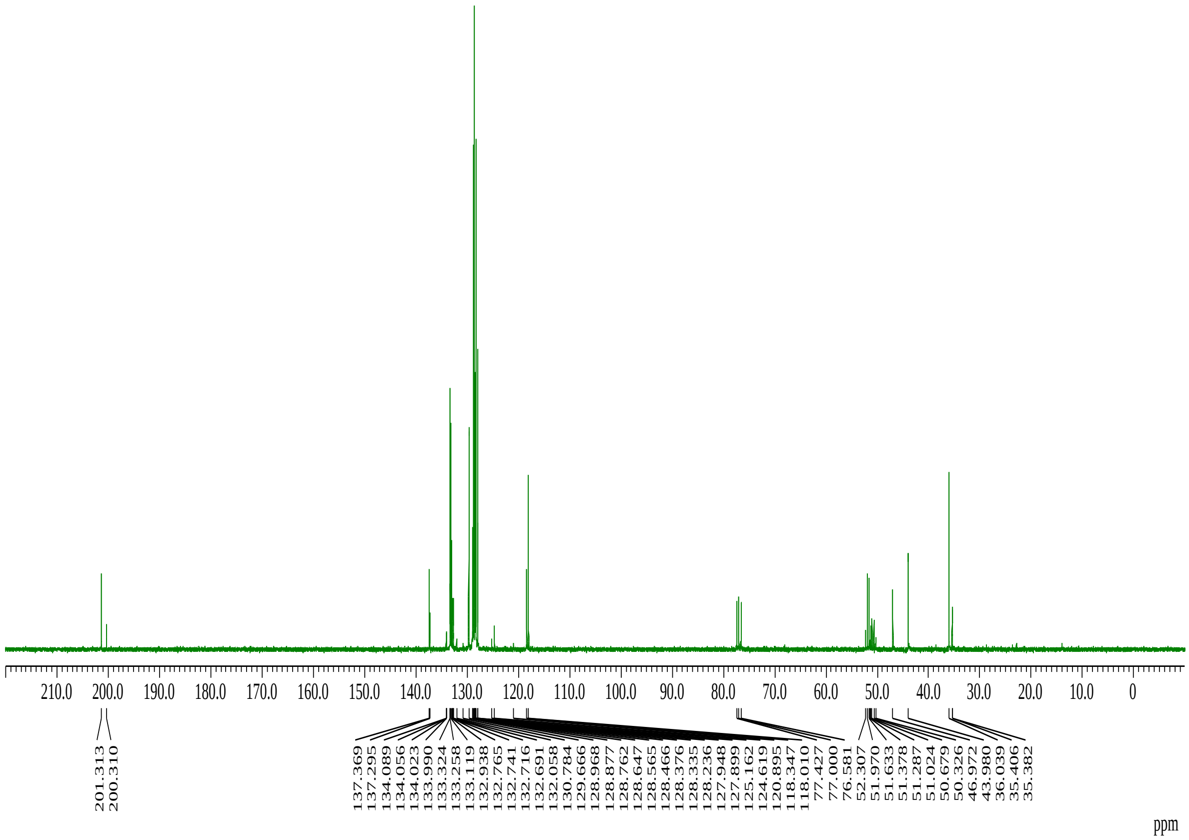


*1.16. 4,4,4-Trifluoro-3-(4-methoxyphenyl)-1-phenyl-2-(prop-2-en-1-yl)butan-1-one (5b)*

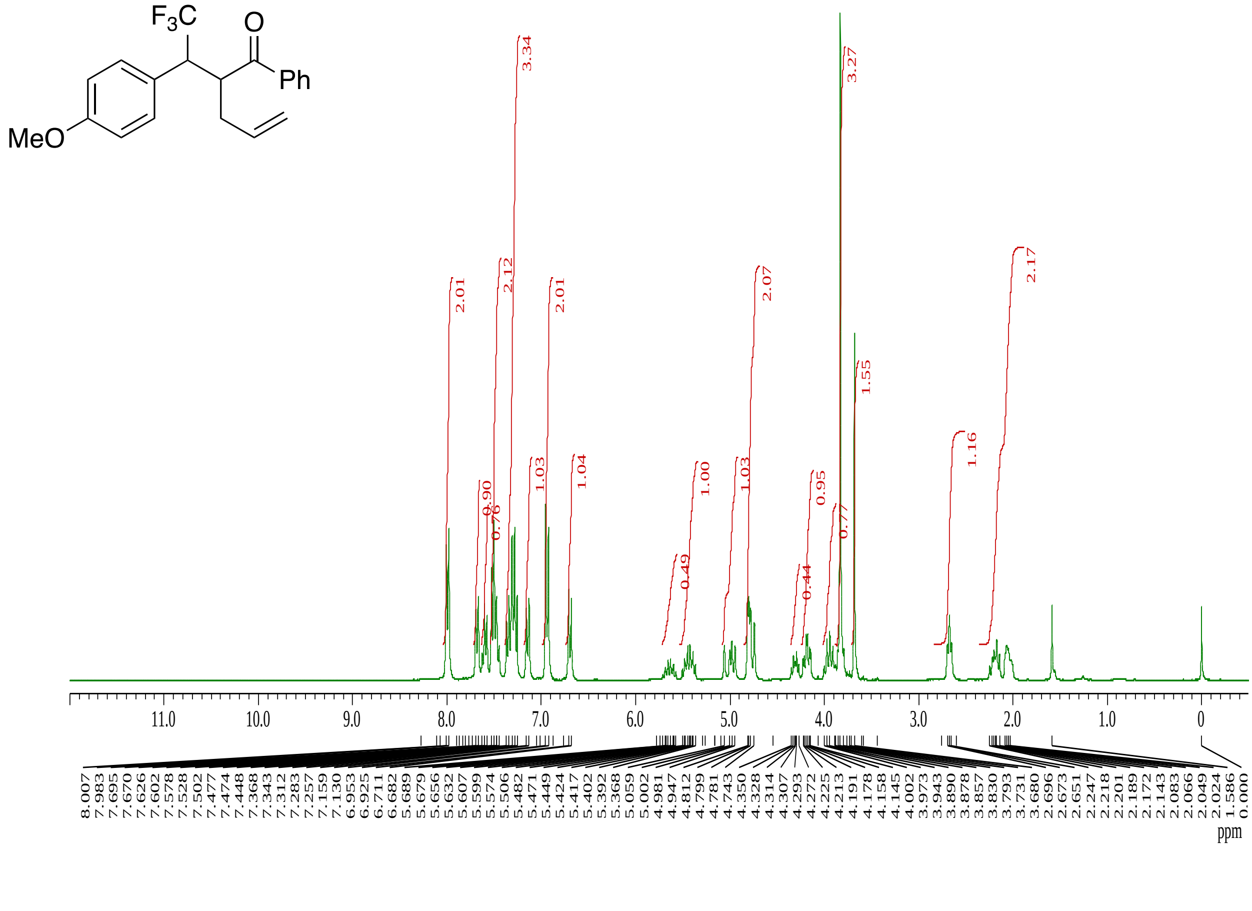

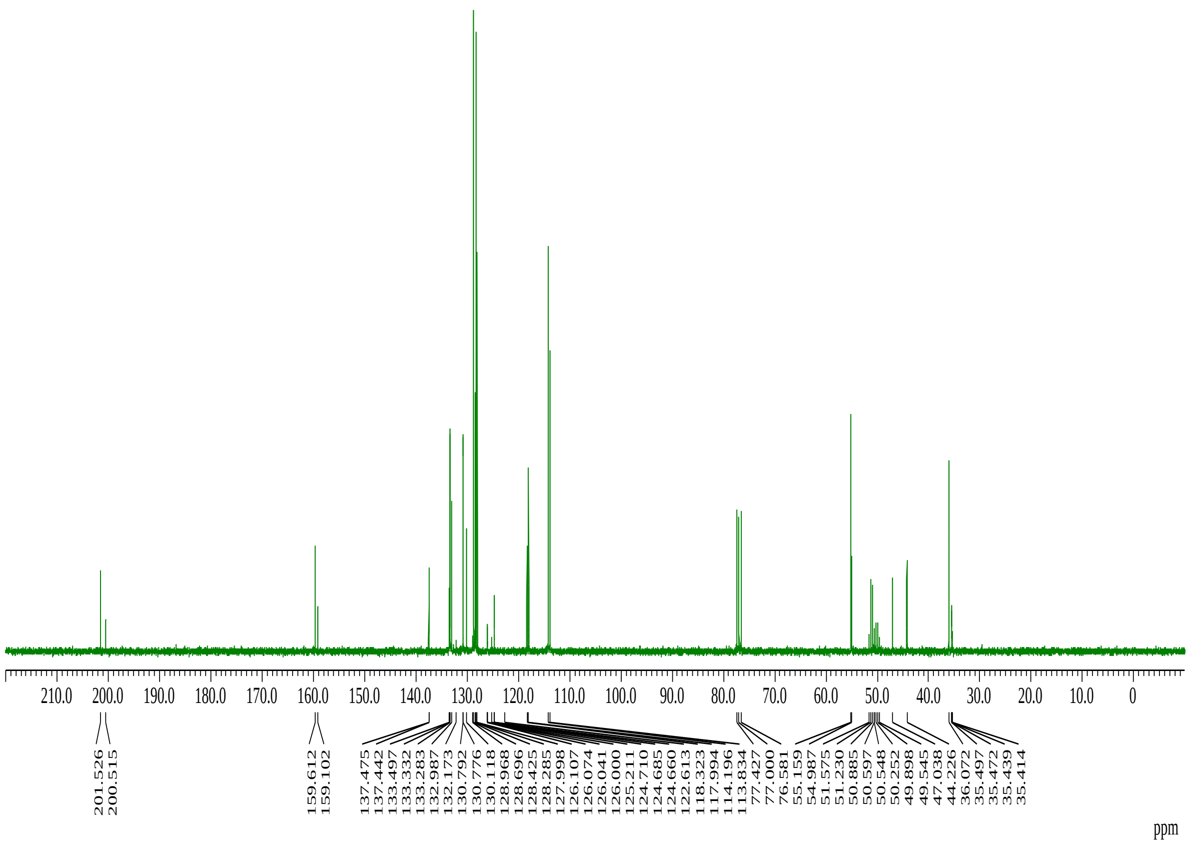


*1.17. 4,4,4-Trifluoro-3-(4-fluorophenyl)-1-phenyl-2- (prop-2-en-1-yl)butan-1-one (5c)*

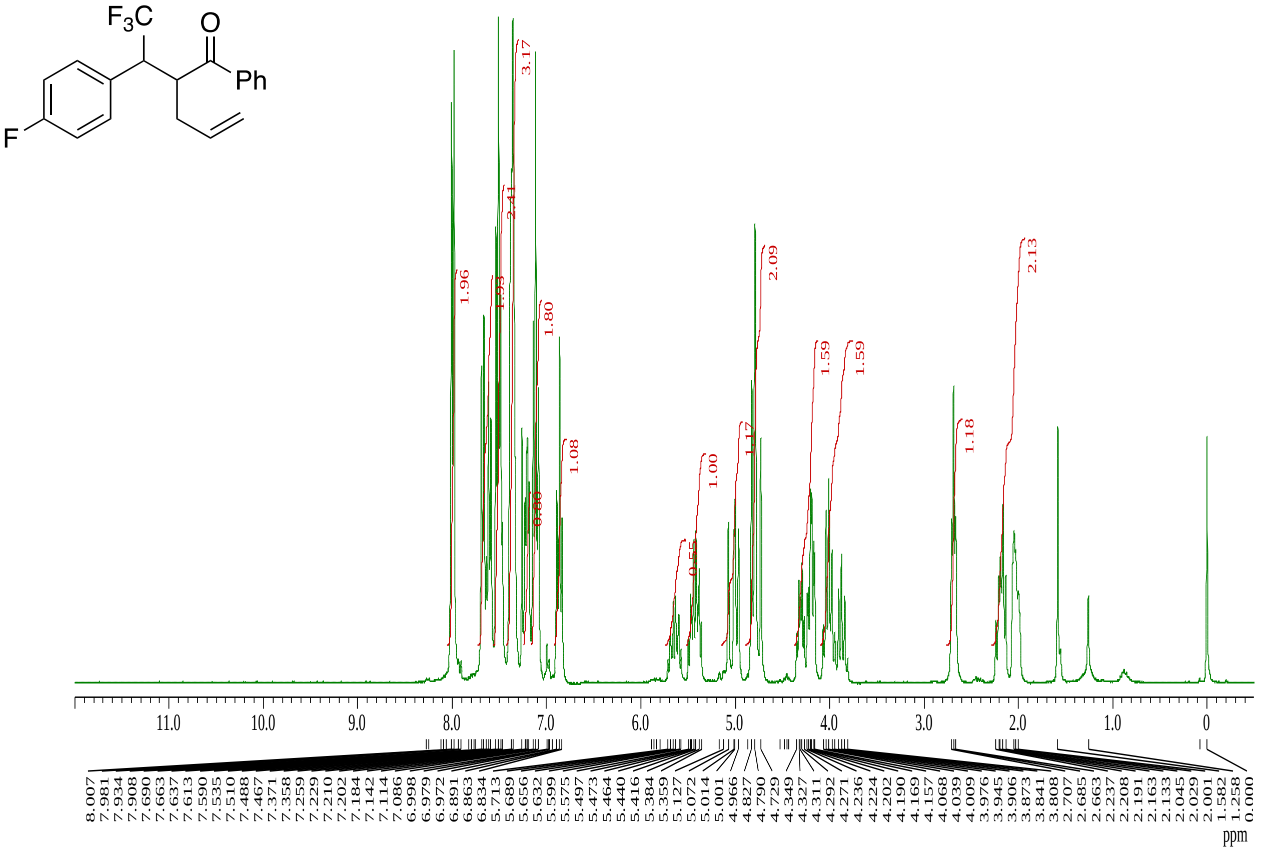

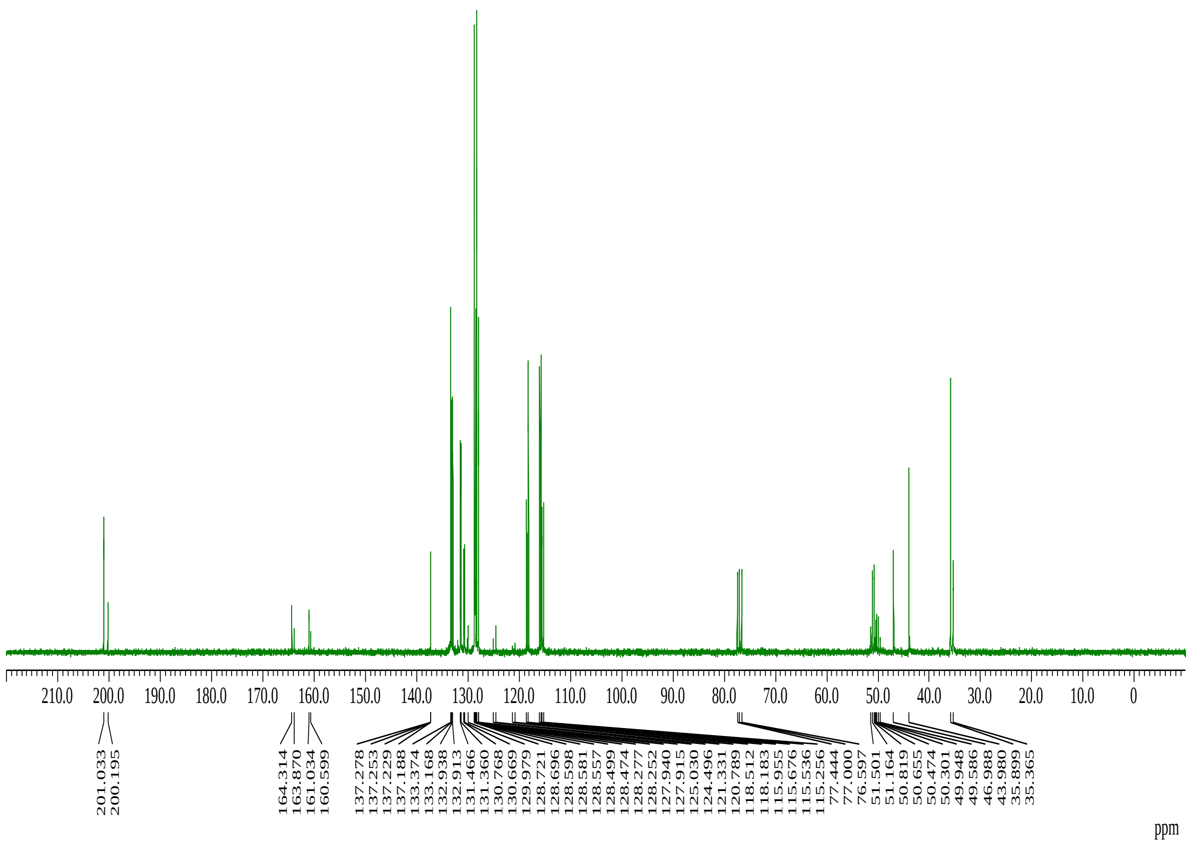


*1.18. 1-Phenyl-2-(prop-2-en-1-yl)-3-(trifluoromethyl)pentan-1-one (5d)*

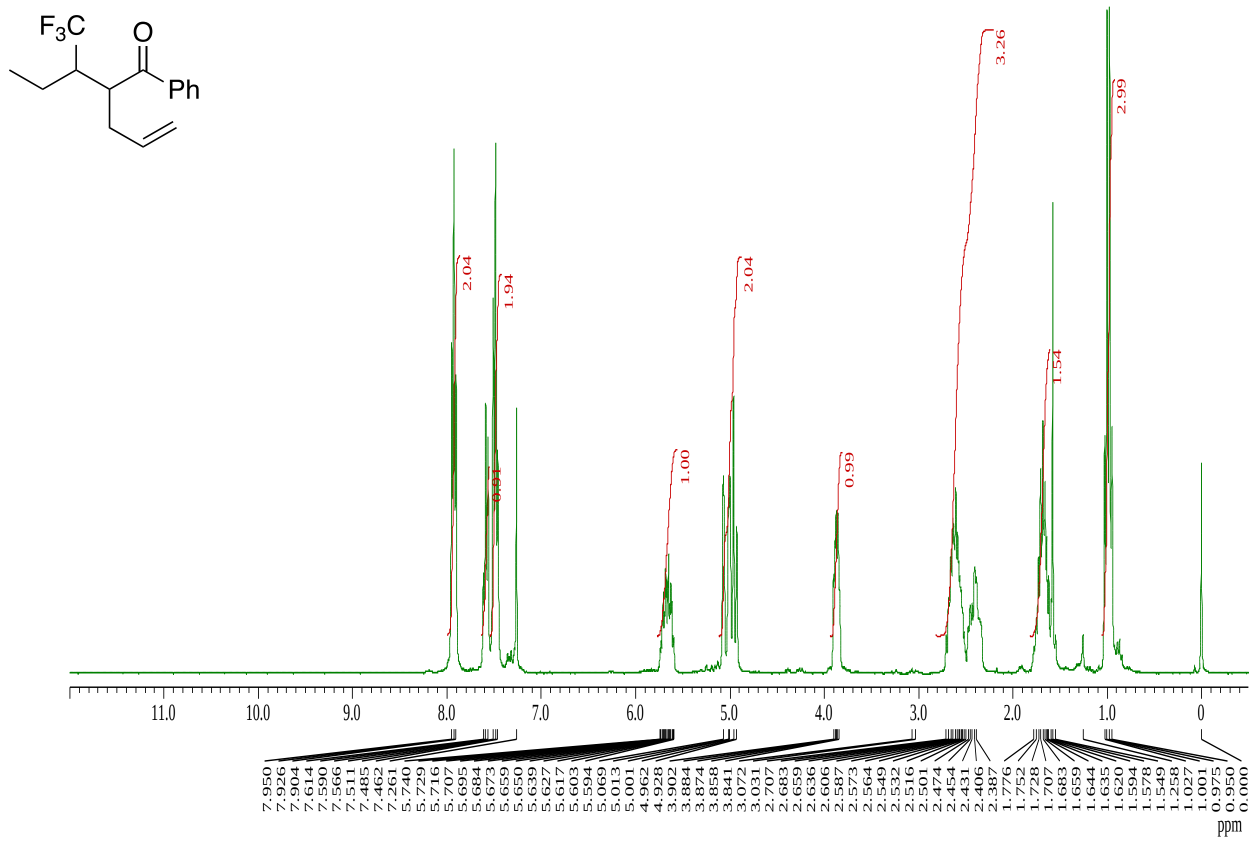

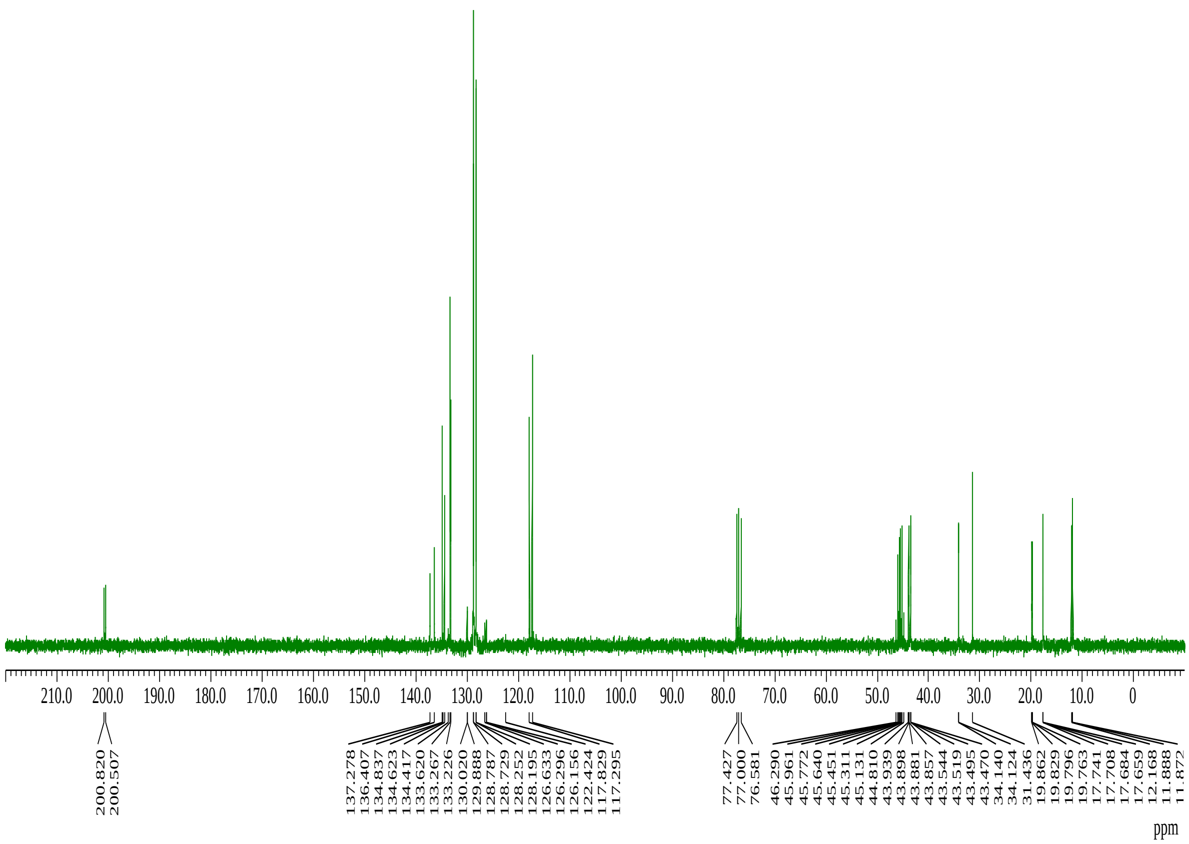


*1.19. 1,5-Diphenyl-2-(prop-2-en-1-yl)-3-(trifluoromethyl)pentan-1-one (5e)*

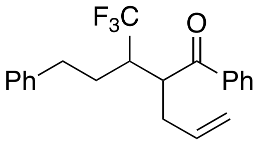


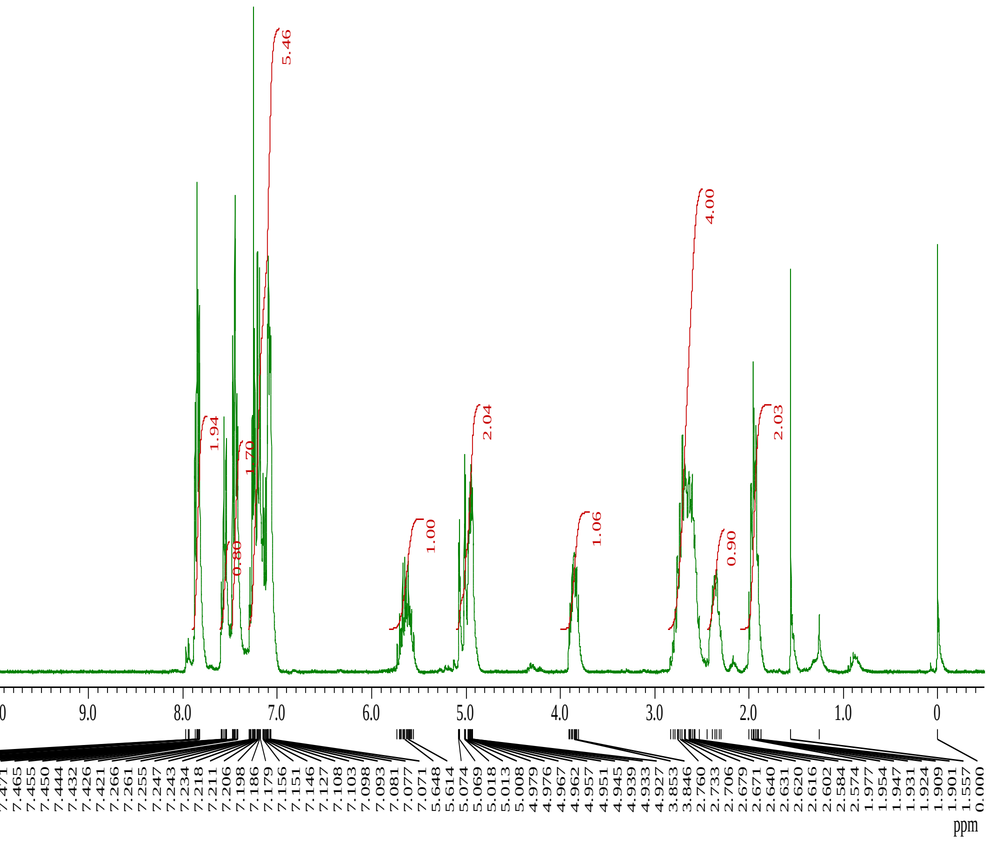

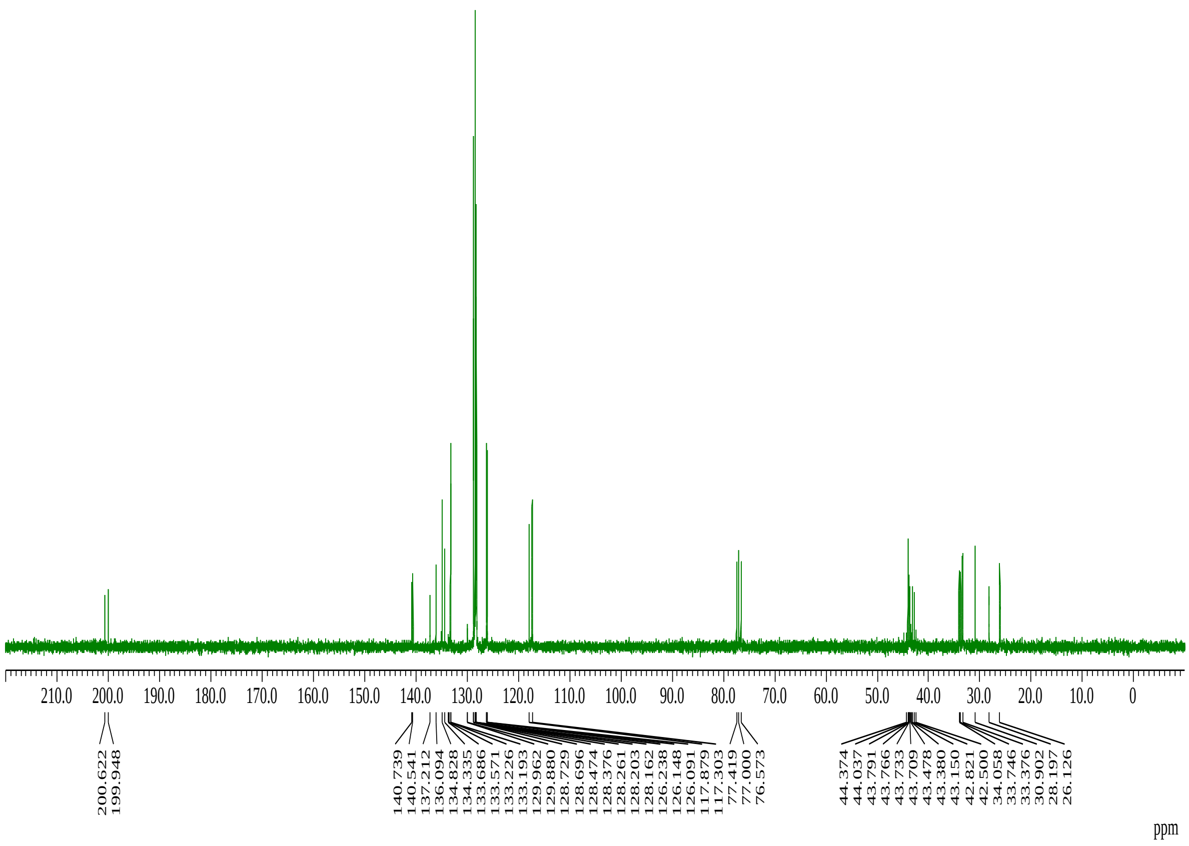

*1.20. 1-(4-Methoxyphenyl)-5-phenyl-2-(prop-2-en-1-yl)-3-(trifluoromethyl)pentan-1-one (5f)*

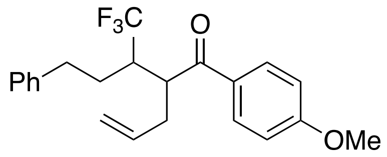

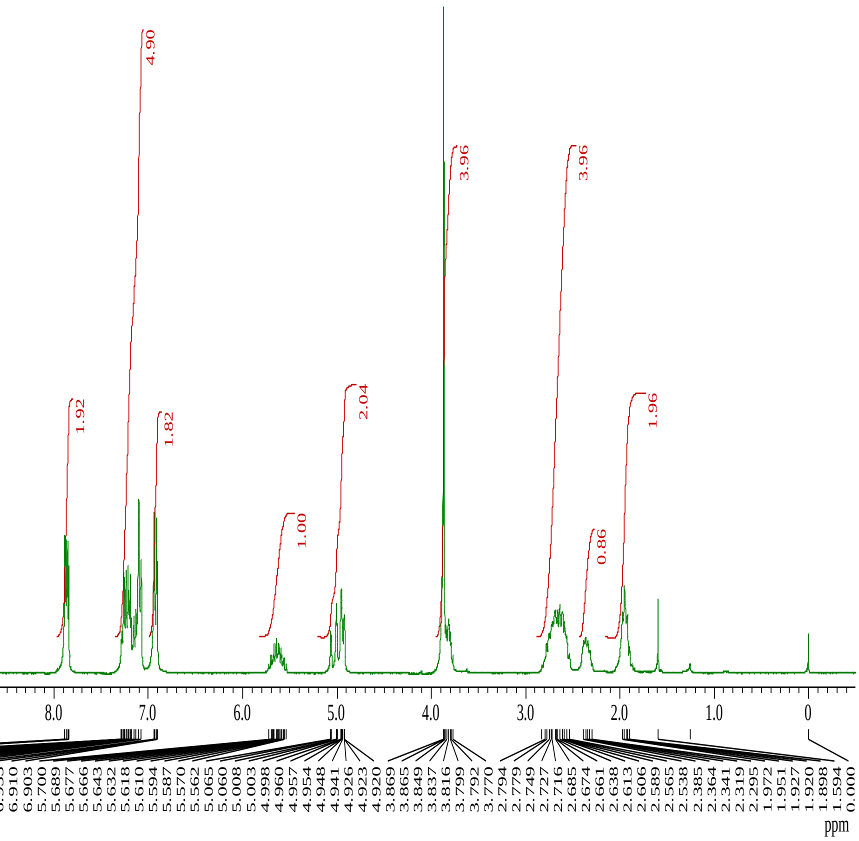

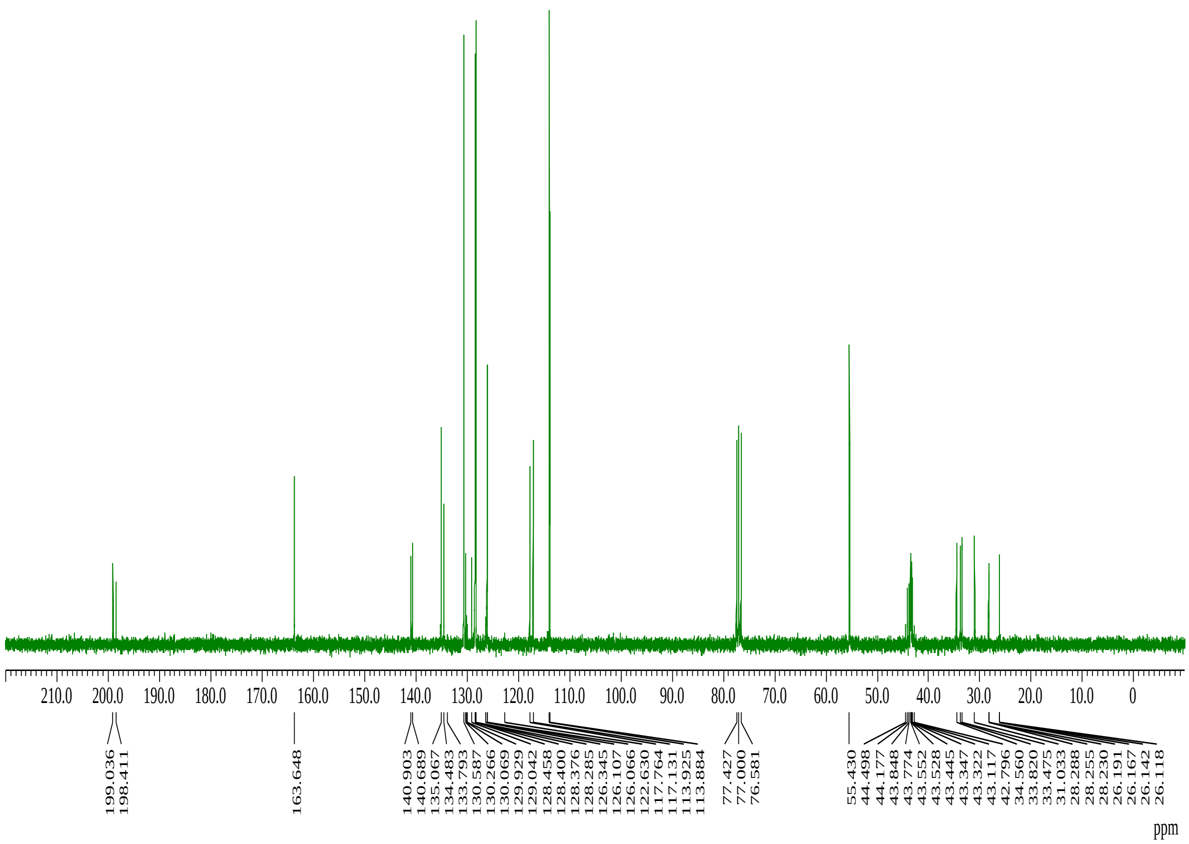


*1.21. 1-(4-Bromophenyl)-5-phenyl-2-(prop-2-en-1-yl)-3-(trifluoromethyl)pentan-1-one (5g)*

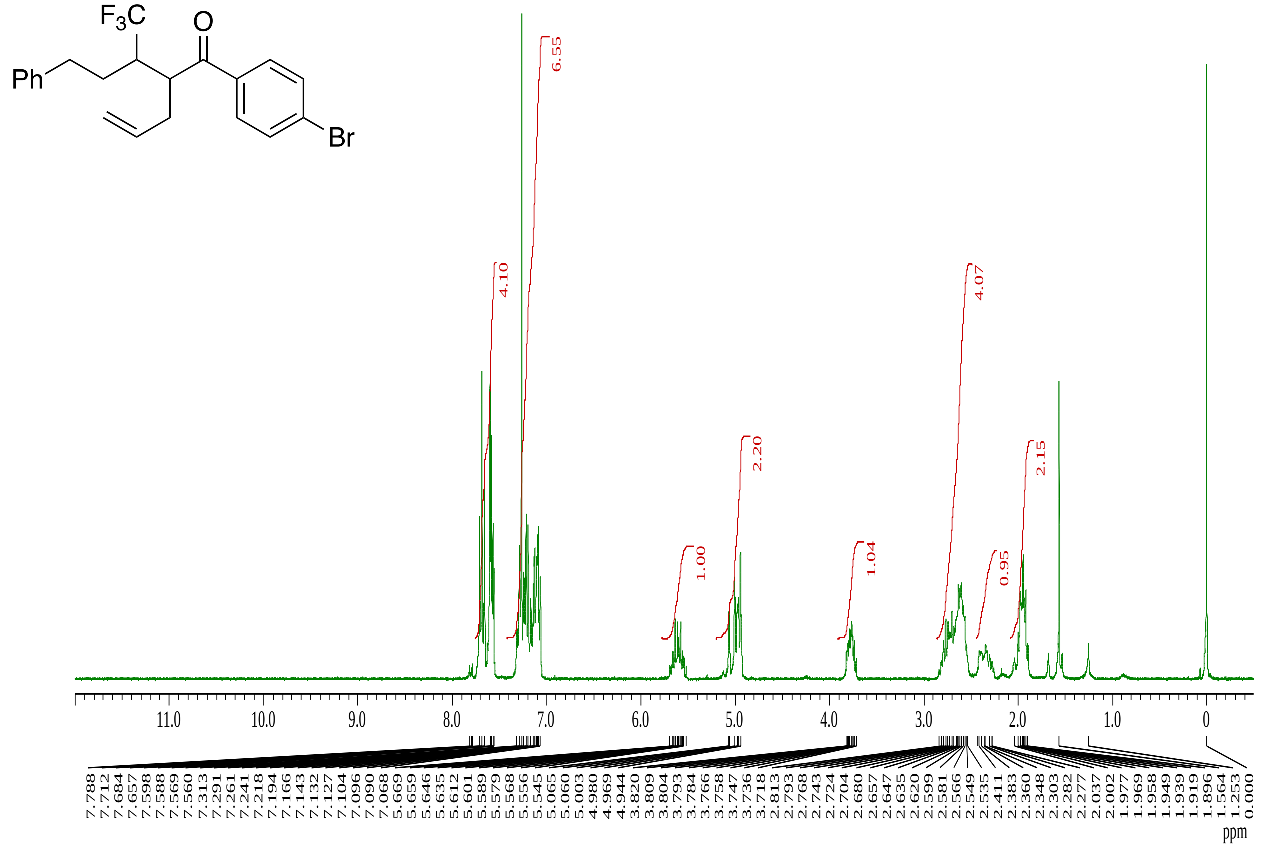

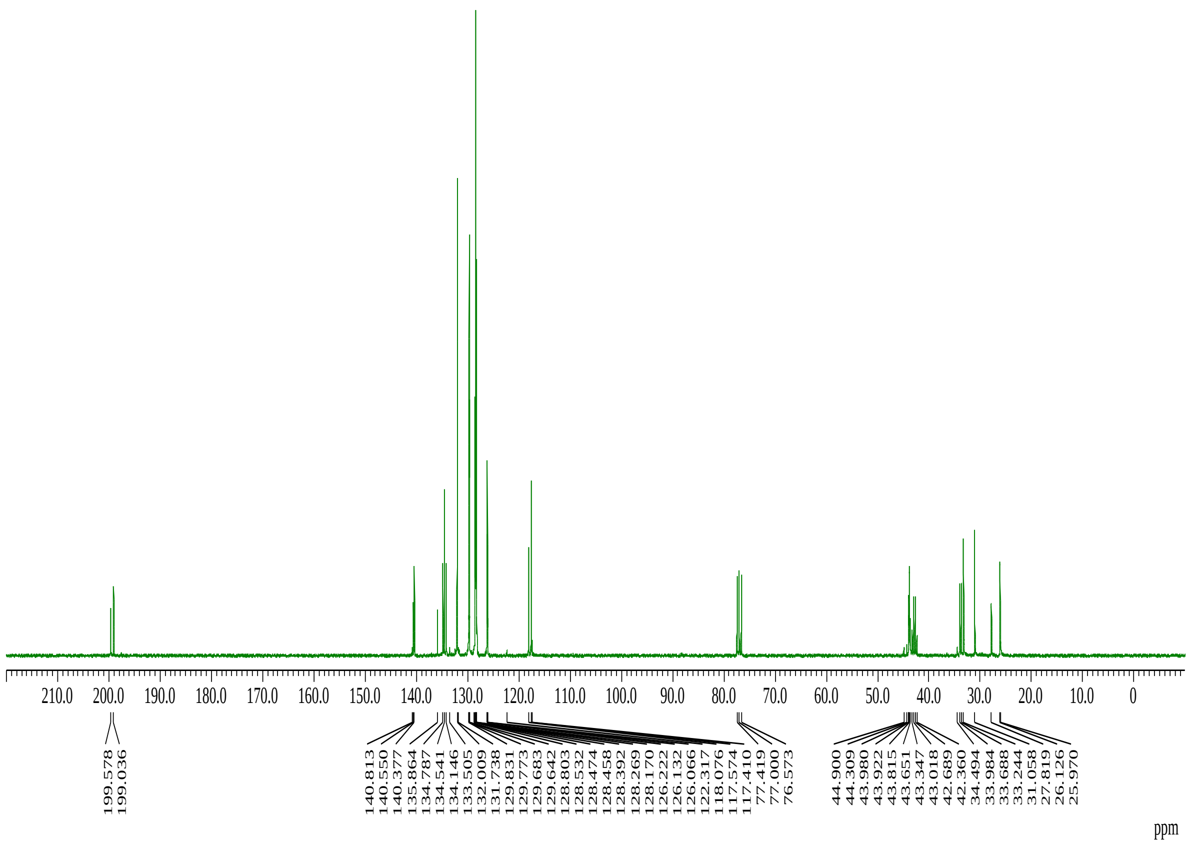
210.0 200.0 190.0 180.0 170.0 160.0 150.0 140.0 130.0 120.0 110.0 100.0 90.0 80.0 70.0 60.0 50.0 40.0 30.0 20.0 10.0 0
ppm
199.578
199.036
140.813
140.550
140.377
135.864
134.787
134.541
134.146
133.505
132.009
131.738
129.831
129.773
129.683
129.642
128.803
128.532
128.474
128.458
128.392
128.269
128.170
126.222
126.132
126.066
122.317
118.076
117.574
117.410
77.419
77.000
76.573
44.900
44.309
43.980
43.922
43.815
43.651
43.347
43.018
42.689
42.360
34.494
33.984
33.688
33.244
31.058
27.819
26.126
25.970
